# Exploration of parents’ and healthcare providers’ perceptions on human milk oligosaccharides (HMOs) and willingness to use HMO-enriched formula: a baseline mixed-methods study from the State of Qatar

**DOI:** 10.3389/fnut.2026.1909697

**Published:** 2026-09-23

**Authors:** Dana Al-Fakhroo, Dalal Hammoudi Halat, Abdel-Salam G. Abdel-Salam, Aaliah Aly, Faisal E. Ibrahim, Nader Al-Dewik, Ahmed Malki

**Affiliations:** 1 Department of Biomedical Sciences, College of Health Sciences, QU Health, Qatar University, Doha, Qatar; 2 QU Health Office of Assessment and Accreditation, QU Health, Qatar University, Doha, Qatar; 3 Department of Mathematics and Statistics, College of Arts and Sciences, Qatar University, Doha, Qatar; 4 Clinical Pharmacy and Practice Department, College of Pharmacy, QU Health, Qatar University, Doha, Qatar; 5 Translational and Precision Medicine Research, Women Wellness and Research Center, Hamad Medical Corporation, Doha, Qatar

**Keywords:** HMO-enriched formula, human milk oligosaccharides (HMOs), perception, Qatar, willingness

## Abstract

**Introduction:**

Human milk oligosaccharides (HMOs) are key bioactive components of breast milk with potential benefits for infant health, immunity, and development. Perceptions of HMOs and willingness to use HMO-enriched formula remain limited in many settings. This study aimed to explore the perceptions of HMOs and willingness to use HMO-enriched formula among parents and healthcare providers (HCPs) in Qatar.

**Methods:**

A convergent mixed-methods design was used. Quantitative data were collected through online, anonymous surveys from parents of children aged 0–2 years and HCPs involved in infant care, to explore perceptions of HMOs and willingness to use or recommend HMO-enriched formula. Qualitative data were obtained through focus group discussions with parents and HCPs to explore further infant feeding experiences, perceptions of HMOs, and views on HMO-enriched formula. Quantitative and qualitative findings were analyzed separately and then integrated.

**Results:**

Among parents, the perception score of HMOs was low, but interest in learning more was high, and willingness to use HMO-enriched formula was generally cautious and conditional. Parents identified HCPs as the most trusted source of information regarding HMOs and HMO-enriched formula, and expressed concerns about safety, evidence, and affordability. Among HCPs, the perception score of HMOs was higher, but formal training was limited and advice about HMO-enriched formula was variable. Qualitative findings showed that both parents and HCPs viewed breastfeeding as the preferred feeding option, while willingness to use HMO-enriched formula depended on clear evidence, safety reassurance, and professional guidance. Integrated findings indicated that future uptake may depend on HCP-led parent education, evidence-based counseling, and improved clinical communication.

**Discussion:**

This is the first exploratory study on HMOs perceptions and willingness to use HMO-enriched formula in Qatar. The findings suggest that perceptions of HMOs and HMO-enriched formula remain variable, and that both parents and HCPs may benefit from clearer, context-appropriate information. The findings indicate that HMO-enriched infant formula use may be associated with multiple practical and informational factors, including professional advice and concerns about safety and evidence. Further research is needed before broader practice implications can be drawn to inform future educational efforts and the integration of HMO-enriched formula into infant nutrition in Qatar.

## 1 Introduction

Early childhood is a crucial stage in human life, during which nutrition has a significant impact not only on short-term physical health but also on future outcomes in immunity, metabolism, and neurodevelopment (1). During this period, the infant intestine is rapidly colonized after birth, with the colonization process shaped by the duration of pregnancy, type of delivery, nature of feeding (breast- or formula-fed), and exposure to medications such as antibiotics. Additionally, the microbiome composition in the infant intestine is associated with immune system maturation (2). During this critical developmental period, breast milk constitutes an essential source of nutrients for infant development. Breastfeeding is always encouraged by international health organizations and healthcare providers; the World Health Organization (WHO) recommends that infants be exclusively breastfed for the first 6 months of life and breastfeeding to be continued until 2 years (3). Studies have shown that human milk is the ideal food for infants for many reasons: it maintains nutrient balance, plays a central protective role against infections, and helps prevent chronic diseases such as obesity, diabetes, and bowel diseases (4). Breast milk is a complete source of nutrients and other bioactive compounds. It contains a high amount of water (5) and other components such as vitamins, minerals, amino acids, fatty acids, hormones, and immunoglobulins, in addition to the main protein and lipid components (6). Among the different bioactive compounds, Human Milk Oligosaccharides (HMOs) are a focus of scientific investigations and product development in infant nutrition (7). Human milk consists of approximately 7.1% of carbohydrates, comprising 80% lactose and 20% HMOs, the latter forming the third most prevalent solid component of human milk, after lactose and fats (8, 9).

HMOs are a family of unconjugated glycans that are produced by lactocytes in the mammary glands (7, 10). The use of HMOs is growing in infant formula, and scientific research has shown their positive effects (11). More than 200 HMOs have been discovered with different structures and compositions (11). Biochemically, HMOs are classified into three major categories based on their composition and functional groups: neutral core HMOs, neutral fucosylated HMOs, and acidic or sialylated HMOs. Each of these categories performs specific biological roles that contribute to the overall health and development of infants (12). For instance, HMOs are identified as essential for the development of the intestinal flora, whereby they can act as a bifidogenic factor, or as natural prebiotics that selectively stimulate the growth of beneficial bacteria (13). Neutral fucosylated HMOs, such as 2′-fucosyllactose (2’-FL), are among the most abundant types of HMOs in human milk, and are fermented by bacteria that predominate the intestinal flora of breastfed infants, producing acetic acid, considered an antimicrobial agent that inhibits harmful microorganisms from adhering to the intestinal mucosa, preventing infection and inflammation (14, 15). Absorption of 2’FL in healthy infants is the same as that of breastfed infants and in infants who are fed with 2’FL-supplemented formula, indicating that the effects of 2’FL and other HMOs exceed the GI tract (16). Furthermore, HMOs have a protective role by anti-adhesive antimicrobial effects, regulating the immune response of intestinal epithelial cells, and directly affecting immune cells and cytokine secretion. Also, some acidic or sialylated HMOs have a positive effect on brain development (17). These HMOs are specifically essential for the development of neurological and cognitive function, with sialic acid being a basic component of brain gangliosides and neural cell membranes (15). Of importance, HMOs should be distinguished from non-HMO prebiotics and probiotics (18). Prebiotics, such as galactooligosaccharides (GOS) and fructooligosaccharides (FOS), have different monosaccharide compositions and structural arrangements, and act as substrates selectively utilized by host microorganisms to confer a health benefit (19). Probiotics, on the other hand, are live microorganisms administered in adequate amounts to confer a health benefit to the host, rather than nondigestible carbohydrate substrates (20).

According to literature, only a small subset of HMOs is currently used in infant formula, which are 2′-FL, Lacto-N-tetraose (LNT), Lacto-N-neotetraose (LNnT), 3′-fucosyllactose (3′-FL) 3′-sialyllactose (3′-SL), and 6′-sialyllactose (6′-SL) (5, 21), as single, binary combinations, or a multi-HMO mixture of five HMOs (5, 22, 23). The health benefits of such additions were previously described, and include supporting the intestinal immune system and gut barrier functions, alongside a shift of the gut microbiome composition closer to that of breastfed infants with higher bifidobacteria (17). In addition, the incorporation of HMOs in infant formula was shown to be associated with lower parent-reported morbidity and less medication use (16). Several health authorities allow the addition of specific HMOs to infant formula; 2’-FL and LNnT were the first two HMO types that were approved as food ingredients, and are safe and well-tolerated, and these supplemented formula contributes to normal growth compared with the standard formula (21, 24). Nowadays, HMO production technologies involve novel processes, which are approved by the regulatory authorities, such as the European Food Safety Agency (EFSA) and the USA Food and Drug Administration (FDA). Both the EFSA in 2015 and the FDA in 2016 approved 2’-FL and LNnT addition to infant formula, and the first formulas containing HMOs were commercialized in Spain and the USA in 2016. The EFSA indicated that the addition of 2′-FL and LNnT at a ratio of 2:1 to infant formula is safe below the age of one year, with maximum doses of 1.2 g/L for 2′-FL and 0.6 g/L for LNnT. In 2019, the FDA stipulated that the maximum dosage of 2′-FL in infant formula is 2.4 g/L, and of LNnT is 0.6 g/L. Moreover, HMOs have obtained the Generally Recognized as Safe (GRAS) status, indicating that they are substances considered safe for their intended use in food by qualified experts. The number of HMOs that can be synthesized on an industrial scale has steadily increased, and currently, formulas containing seven HMOs (2’-FL, 3´-FL, LDFT, LNnT, LNT, 3’-SL, 6’ − SL) are being studied (25).

Despite these benefits and advances, consumers’ perceptions and willingness to use HMO-enriched infant formula remain very scarcely investigated. A recent focus group investigation on patients’ perspectives on HMO use was done (26); yet, the study did not assess HMO perceptions regarding infant nutrition. Moreover, although several international regulatory bodies have approved the incorporation of HMOs into infant formula and their safety, it remains unclear how parents and healthcare providers perceive HMO-enriched formula, its benefits for infants’ health, and their willingness to use it, and this gap of knowledge exists for the state of Qatar. The high standard of healthcare in Qatar and the country’s multicultural population provide a distinctive environment for investigating parents’ perceptions and behaviors regarding infant feeding. As such, studying how parents in Qatar respond to and perceive innovations such as HMO-enriched formula is well-timed and critical to ensuring that public health policy and industry practices are grounded in the population’s needs and beliefs. Having HMO-enriched formula in the Qatari market offers an alternative to human milk for mothers who are not fully breastfeeding. We hypothesize that perceptions and understanding of HMO-enriched infant formula among parents and healthcare providers (HCPs) are heterogeneous and not well characterized, and that these perceptions may be associated willingness to use such products (27). The absence of evidence regarding parental and healthcare professional perceptions toward HMO-enriched formula may impede effective implementation of emerging infant nutrition strategies and limit the ability of healthcare systems to address barriers to acceptance and utilization of these products.

This study, therefore, aims to explore perceptions toward HMOs and willingness to use HMO-enriched infant formula among HCPs and parents in Qatar, through both a quantitative measurement of perceptions and willingness to use these compounds, and a qualitative, in-depth exploration. This study remains situated within a “breastfeeding-first” conceptual framework, consistent with the WHO and UNICEF global strategy to protect, promote, and support breastfeeding. Within such framework, human milk is recognized as the gold standard for infant nutrition and the primary natural source of HMOs (28, 29). As such, the exploration of HCPs’ and parents’ perceptions of HMOs and willingness to use HMO-enriched infant formula in this study is not intended to promote formula use, but to better understand gaps and contextual factors that may be associated with infant feeding practices. The study aims to get insights that can be incorporated into conversations surrounding infant nutrition, support guidance for feeding recommendations, and supplement the educational campaigns directed toward parents.

## 2 Methods

### 2.1 Study design, setting, and participants

The study used a convergent mixed-methods design to explore the perceptions and willingness to use HMO-enriched formula among parents and HCPs in Qatar, with separate collection and analysis of quantitative and qualitative data, followed by later integration of findings. The study was conducted collaboratively between the Women Wellness and Research Center (WWRC) at Hamad Medical Corporation (HMC), the public health facility of the State, and Qatar University (QU). For participant recruitment in both the quantitative and qualitative phases, licensed HCPs from HMC relevant departments that provide care for infants aged 0–2 years were approached, including those from pediatrics departments, nutrition department, breastfeeding department, NICU, etc. All HCPs who were not experienced in infant care or nutrition were excluded. For participant recruitment among parents, and also in both study phases, the Principal Investigator approached parents of children up to 5 years; yet, and in line with the WHO recommended periods for breastfeeding, analysis was limited to parents who had children aged 0–2 years. The study was conducted in accordance with the principles of the International Code of Marketing of Breastmilk Substitutes (30) and relevant World Health Assembly resolutions (31), whereby no breastmilk substitutes were advertised or promoted, no formula manufacturers or commercial distributors were involved in the design, funding, data collection, analysis, or interpretation of this study. Furthermore, no branded materials, manufacturer logos, complimentary samples, or promotional content related to infant formula were used during any phases of recruitment, consent, or data collection.

### 2.2 Quantitative method

#### 2.2.1 Sampling

A representative sample size from both HCPs and parents was determined using Raosoft® online calculator. The minimal sample size for HCP was 109 and for parents was 385. Participants were recruited using a convenience sampling approach. Eligible parents and healthcare providers who were accessible during the study period and agreed to participate were included in the study.

#### 2.2.2 Survey development

Three anonymous surveys were used for the quantitative data collection, two for parents (Arabic and English versions) and one for HCPs in English. The parent survey was developed in both languages to consider that some mothers were uncomfortable answering it in English. Survey questions were based on a comprehensive literature review and prior studies in the related field (32–36). As no validated instruments currently exist for the Qatari or Arabic-speaking context, the surveys were pilot tested with a small group (5 participants from each target population) to assess clarity, cultural appropriateness, and content relevance. Suggested modifications as a result of the pilot were then used to adjust the surveys and the piloting responses were excluded from the analysis.

A total of 26 questions were included in the parents’ survey, and 29 in the HCPs’ survey. The surveys began with an introductory disclaimer that emphasized human milk as the gold standard for infant feeding, and that the purpose of the research was to contribute to scientific understanding of infant feeding, not to promote the use of breastmilk substitutes. Further, the surveys’ language was neutral and non-promotional, and did not imply that HMO-enriched formula is equivalent to human milk. The surveys referred to “infant formula” and “HMO-enriched formula” in generic, non-branded terms and did not include any brand names or images of commercial products. The introductory statements also included the study purpose, ethical approval, risks, benefits, duration, and confidentiality policy in simple terms. Participants were also informed that participation is voluntary, not associated with benefits or compensation, and that they have the freedom to withdraw from the survey at any time if they decide that. Both surveys were divided into 4 sections with a fixed order, starting with (1) sociodemographics, then (2) HMO perceptions along with true/false knowledge statements on their properties, (3) HMO-enriched formula perceptions, and ending with (4) future willingness to use HMO-enriched formula. The parents’ survey explored breastfeeding practices, infants’ nutrition choices, perceptions of HMOs and HMO-enriched formula, and willingness to use such formula for their infants’ nutrition. The HCPs survey investigated their perceptions of HMOs and HMO-enriched formula, their clinical practice regarding HMO-enriched formula, and their willingness to recommend such formula as part of nutrition for infants, as well as the associated challenges that could affect such willingness. Questions on baseline perception of HMOs (e.g., “Have you ever heard about human milk oligosaccharides (HMOs)?” and related true/false statements) were presented before items on willingness to use HMO-enriched formula, in order to capture spontaneous, uninformed perceptions. No promotional statements or educational descriptions of HMOs benefits were provided between sections, and brief, neutral wording was used in the willingness items (e.g., “Do you think you will use HMO-enriched formula?”). As a result, willingness questions primarily reflected spontaneous responses following baseline perception exploration, with minimal informational prompting, rather than responses to a detailed explanatory survey.

The structure of the response in both surveys was based on multiple-choice questions, for sociodemographics, HMO perception, HMO-enriched formula perception, and future interest. A dichotomous perception score composed of 7 true/false items was used to explore parents’ and HCPs’ information about HMOs and their benefits, and open-ended, optional questions were included to collect additional explanations regarding perceived outcomes and safety concerns.

#### 2.2.3 Data collection

The survey form was sent to participants via an invitation email and personal communication, briefly describing the study objectives and including a link to the online survey, developed through the free survey tool in Google Forms. Participants recruitment was independent of commercial interests, and no incentives linked to infant formula companies or other beneficiaries were provided. The HCPs emails included the link to the HCP survey and the parent email included the link to the parent survey. Upon clicking the link, potential participants were presented with a consent statement to inquire about their agreement to participate in the study. No physical consent form was signed. Instead, participants indicated their agreement to participate in the study online by selecting “I consent to participate” in order to access the survey questions. Recruitment of participants was conducted from August 2025 to January 2026. The average time required to complete the survey was approximately 10 min. Of the 150 invited HCPs, 61 consented to participate, 3 refused, and the remainder did not respond to the invitation. Of the 400 invited parents, 291 consented to participate, 102 participants were excluded as their children were aged over 2 years, 9 refused, and the remainder did not respond.

#### 2.2.4 Data analysis

The Arabic parent dataset from the parent surveys was translated into English and merged with the English version to create a single harmonized parent dataset, as both versions included identical items measuring the same constructs. Data preparation and statistical analysis were conducted using R (37), selected for its reproducibility, transparency, and ability to handle multilingual data cleaning and generate publication-quality outputs. Prior to analysis, column names were standardized to ensure compatibility with R syntax, and only responses from participants who provided explicit consent were retained. Missing data were handled item-by-item: percentages were calculated using non-missing responses as denominators, and complete-case analysis was applied for each inferential test, retaining only observations with non-missing values for all variables involved in that specific test. Categorical variables (e.g., age group, nationality, education level, number of children, awareness, and behavioral responses) were converted into factor variables. Perception-related items were recoded into binary variables (correct versus incorrect). Accordingly, an exploratory HMO perception index was constructed from the seven factual true/false items. Each correct response received one point, producing a total score ranging from 0 to 7. Because the items were designed to assess several distinct domains of HMO-related, heterogeneous factual knowledge, rather than a single latent construct, the index was intended solely for descriptive and exploratory analyses and was not considered a validated psychometric scale. This perception score is exploratory and non-validated, and all interpretations based upon the total score are made with appropriate caution and considered exploratory in nature.

Assumptions for statistical testing were evaluated prior to analysis. Normality of the perception score was assessed using the Shapiro–Wilk test and was found to be violated in both the parent dataset. Homogeneity of variance between the primary comparison groups was assessed using the F test (var.test in R) and was not violated in either dataset. Given the non-normal distribution and bounded nature of the perception score (range 0–7), non-parametric tests were selected for all group comparisons involving this variable.

The Mann–Whitney U test was used to compare perception scores between exactly two independent groups. This non-parametric test ranks all observations and assesses whether one group tends to have systematically higher ranks than the other; it is appropriate for ordinal or non-normally distributed continuous outcomes. The Kruskal–Wallis test was used when perception scores were compared across three or more independent groups. This test is the non-parametric equivalent of one-way ANOVA and evaluates whether at least one group distribution differs meaningfully from the others.

Associations between two categorical variables were examined using Pearson’s chi-square test. Fisher’s exact test was applied instead, particularly in 2 × 2 tables, to ensure valid inference. Spearman’s rank correlation coefficient was used to quantify the strength and direction of monotonic relationships between ordinal or non-normally distributed variables, including the perception score, perceptions, and willingness to use or recommendation. Spearman’s correlation makes no distributional assumptions and is computed from the ranked values of the observations.

Because this study employed a cross-sectional observational design, all regression analyses were considered exploratory and intended to quantify statistical associations rather than establish causal or predictive relationships. Furthermore, to account for multiple hypothesis testing, *p*-values from the primary inferential analyses were adjusted using the Benjamini-Hochberg false discovery rate (FDR) procedure where applicable.

### 2.3 Qualitative method

#### 2.3.1 Sampling

Participants were recruited via invitation emails sent by the Principal Investigator from the HMC Outlook address book for HCPs and through personal communication for mothers. Both populations were initially approached during quantitative data collection. The emails briefly introduced the study objectives and inquired about willingness to participate in focus groups to further explore perceptions and willingness to use or recommend HMO-enriched formula. Twelve HCPs, including nutritionists, breastfeeding specialists, pediatricians, and midwives, participated in 3 separate FG. Also, 9 mothers of infants less than 2 years old participated in 2 separate FG.

#### 2.3.2 Data collection

Discussions were conducted in English for HCPs and in Arabic for parents based on participants’ fluency. Consent was obtained from participants by signing a consent form in a digital PDF before each session to indicate their voluntary willingness to participate in the study and their agreement to record the FG discussions. Participant confidentiality was strictly maintained throughout the study, whereby each participant was assigned a unique code for use in transcripts and analysis to prevent identification. Data access was restricted only to the Principal Investigator and the research team.

A semi-structured set of questions was developed for each group based on the study objectives and used to guide the FG discussions. As for surveys, the FG questions referred to generic “infant formula” and “HMO-enriched formula” without any commercial brands, and no incentives were provided to participants. Questions were intended to deeply explore parents’ and HCPs’ insights regarding infants’ feeding practices, HMOs’ perceptions, and willingness to use HMO-enriched infant formula. Each FG lasted approximately 30–60 min. All sessions were audio-recorded for transcription. Once each FG started, no participants dropped out. The Principal Investigator moderated all the FGs to ensure consistency. FG discussions were stopped when no new ideas emerged, indicating that further data collection through FG was unnecessary. Data saturation was evaluated primarily from the researchers’ perspective: discussions within each participant group (parents and HCPs) were considered to have reached saturation, and no further groups were scheduled, once successive sessions ceased to yield new codes, themes, or subthemes. This was corroborated from participants’ perspective, in that participants had no further insights to add once a given topic had been discussed (38).

#### 2.3.3 Data analysis

Audio recordings were transcribed verbatim. For sessions conducted in Arabic, transcripts were translated into English by a certified translator and revised against the original Arabic text to confirm correctness and maintain the spirit of the discussion. The transcribed data was analyzed thematically using NVivo software (version 15) to determine common patterns and themes. Thematic analysis was conducted by a researcher experienced in qualitative data analysis, starting by repeated readings of the transcripts and listening to audio-recordings to become familiar with the data, while recording analytical notes to inform subsequent coding. An inductive approach was used during the analysis to identify ideas related to HMOs, and the key points that emerged during the discussion helped clarify HCPs’ and mothers’ perceptions and willingness for recommending or using HMO-enriched formula. Transcripts were systematically coded and categorized into themes and sub-themes (39, 40).

Coding was led by one researcher (DA), who generated the initial code list and thematic structure for each participant group directly from the transcripts. All coded transcripts and the resulting themes were subsequently read and independently reviewed by two additional co-authors (AA, DHH), who assessed whether the codes and themes were adequately grounded in participants’ accounts. This reflects a two-perspective, team-based coding process, comprising one primary coder and an independent second-level review, consistent with recommended practice for enhancing confirmability and dependability in applied qualitative research (41). Differences in interpretation identified during this review were resolved through discussion among the research team until consensus was reached; no disagreements required third-party arbitration. As the thematic analysis followed a reflexive approach, coding consistency was pursued through this collaborative review and consensus process rather than through calculation of a quantitative inter-rater reliability statistic, consistent with recommended practice for reflexive thematic analysis (42). The analytic process was iterative, with codes and themes reviewed and refined repeatedly to ensure that the final themes accurately reflected participants’ experiences. Representative quotes from participants were selected to illustrate each of the identified themes.

Trustworthiness of the qualitative finings was established through measures described by Lincoln and Guba’s framework (43). Credibility was supported by the use of triangulation with quantitative data. Although member checking was not conducted due to participants being unavailable for follow-up, several strategies were employed to ensure credibility and accuracy of the qualitative findings. These included the use of verbatim participant quotations, systematic coding procedures, collaborative review of themes by the research team, and triangulation with quantitative findings. Dependability was addressed through the maintenance of a structured codebook and illustrative exemplars, ensuring a consistent and documented analytical trail. Confirmability was supported through researcher reflexivity: team members explicitly acknowledged their positionality as healthcare professionals and educators with potential investment in the course outcomes. Transferability was supported through the provision of description of the study context, participant characteristics, and representative quotes illustrating each of the identified sub-themes.

## 3 Results

### 3.1 Quantitative results

#### 3.1.1 Parent survey results (combined Arabic and English surveys)

A total of 300 parent responses were collected. Of these, 291 (97.0%) provided consent. Following application of the inclusion criterion (youngest child aged 24 months or younger), 102 respondents (34.0%) were excluded, yielding a final analytic sample of 189 parents from both language versions. Participants were mainly young-to-middle-aged, mostly Qatari, and highly educated. Breastfeeding was common, with 91.0% of eligible parents reporting previous breastfeeding. Current feeding practices showed a mixed pattern with 45.5% partially breastfeeding and 28.0% exclusively breastfeeding. The full demographic details of participants are shown in Table 1.

**Table 1:** Sociodemographic characteristics of parent participants.

Characteristic	Category	(%)
Age group	20–29 years	89 (47.1%)
	30–39 years	79 (41.8%)
	40–45 years	15 (7.9%)
	>45 years	6 (3.2%)
Nationality	Qatari	138 (73%)
	Non-Qatari	51 (27%)
Education	Primary school	0 (0%)
	Middle school	3 (1.6%)
	High school	22 (11.6%)
	Bachelor’s degree	135 (71.4%)
	Master’s degree	24 (12.7%)
	Doctoral degree	5 (2.6%)
Number of children	1	51 (27%)
	2	47 (24.9%)
	3	41 (21.7%)
	4+	50 (26.4%)
Youngest child age	0–6 months	81 (42.9%)
	6–12 months	54 (28.6%)
	12–18 months	32 (16.9%)
	18–24 months	22 (11.6%)
Parents breastfeed previously	Yes	172 (91%)
	No	17 (9%)
Parent current nutrition	Partial breastfeeding with formula	86 (45.5%)
	Exclusive breastfeeding	53 (28%)
	Exclusive formula feeding	50 (26.5%)
Parent perception and willingness to use summary	Heard about HMOs-Yes	36 (19%)
	Heard about HMOs-No	121 (64%)
	Heard about HMOs-Not sure	32 (16.9%)
	Heard about HMO-enriched formula-Yes	32 (16.9%)
	Heard about HMO-enriched formula-No	128 (67.7%)
	Heard about HMO-enriched formula-Not sure	29 (17.3%)
	Will use HMO-enriched formula-Yes	35 (18.5%)
	Will use HMO-enriched formula-Possibly	118 (62.4%)
	Will use HMO-enriched formula-No	36 (19%)
	Interested in knowing more-Yes	172 (91%)
	Interested in knowing more-No	17 (9%)
Parent perception item answered correctly	Natural components in human milk	173 (91.5%)
	Available in infant formulas	75 (39.6%)
	Destroyed by stomach acidity (correct = no)	98 (51.8%)
	Supports good bacteria in the gut.	166 (87.8%)
	Important for immunity	171 (90.4%)
	Reduces allergic reactions	154 (81.4%)
	Negative brain effect (correct = no)	126 (66.6%)

Percentages based on non-missing responses.

Most parents had not heard of HMOs (64.0%) and HMO-enriched formula (67.7%), while 19.0 and 16.9% have heard of HMOs and HMO-enriched formula, and 16.9 and 15.3% were unsure, respectively (Figure 1). Regarding willingness to use HMO-enriched formula, 62.4% indicated “possibly,” 18.5% said yes, and 19.0% said no (Figure 2), indicating moderate uncertainty.

**Figure 1:**
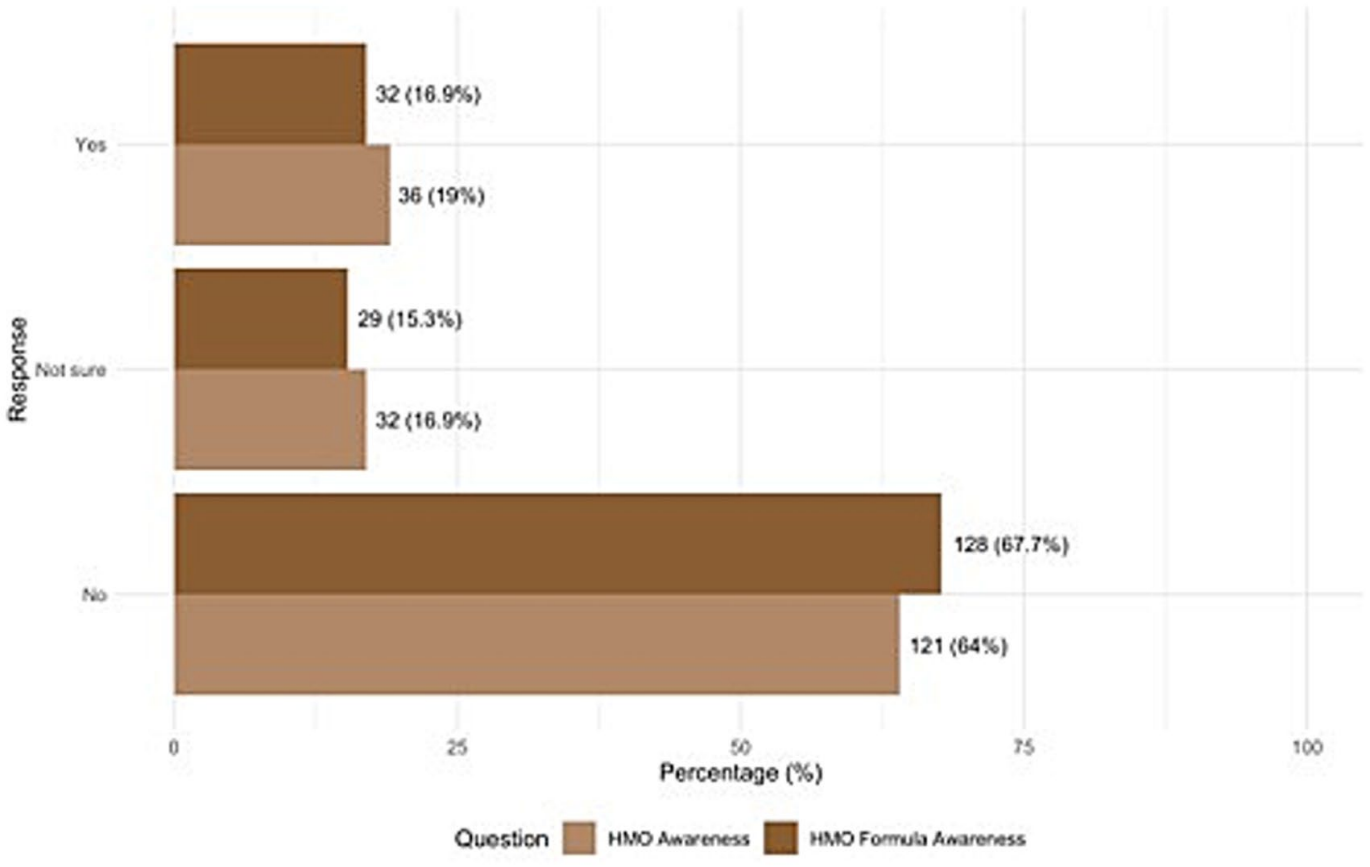
Distribution of parents regarding whether they heard or not of HMOs and HMO-enriched formula (*n* = 189).

**Figure 2:**
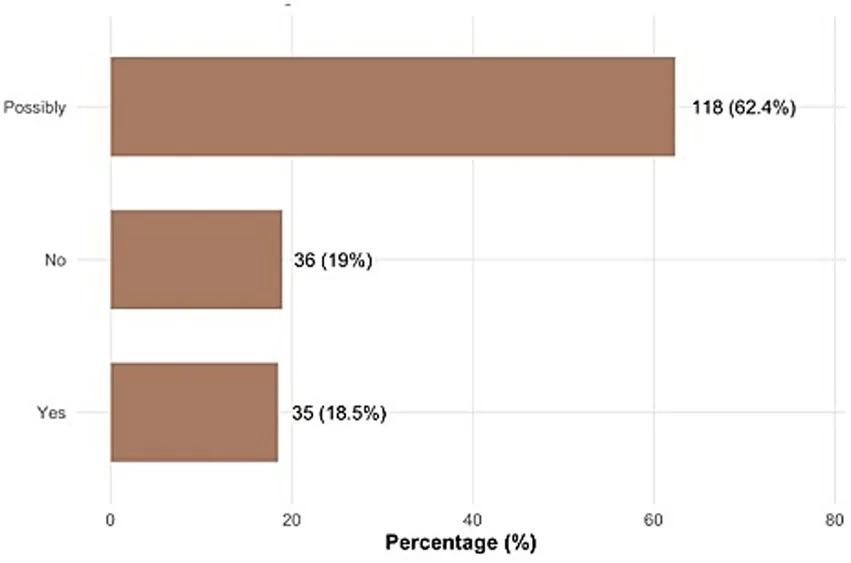
Distribution of parents’ willingness to use HMO-enriched formula (*n* = 189).

Table 2 shows that parents were generally positive toward HMOs, yet cautious. A substantial proportion agreed (33.9%) or strongly agreed (30.2%) that HMO-enriched formula should exist in Qatar. Recommendation behavior was similarly cautious, with 43.4% willing to recommend and 49.7% unsure. Reports of HCPs’ recommendations were limited: the majority of parents indicated that their HCPs did not recommend an HMO-enriched formula (24.3%) or were unsure whether it was recommended (64.0%), highlighting a possible clinical communication gap. Despite this, interest in learning more about HMOs was very high (91.0%), reflecting curiosity and receptiveness to new information. Regarding feeding preferences, 47.6% reported no specific formula preference, while 36.0% preferred HMO-enriched formula. The presence of HMOs in formula played a role in decision-making among some parents, with 34.4% indicating they would be more likely to choose such a formula. HCPs were identified as the most trusted information source (36.5%), emphasizing their central role in shaping parental perception. Overall, while perceptions of HMOs remain limited, there was a willingness to learn and a conditional acceptance of an HMO-enriched formula among eligible parents. Perceptions of HMOs, measured using the seven-item score, varied across items. High proportions correctly identified HMOs as natural components of human milk (91.5%), their role in supporting gut bacteria (87.8%), and their importance for immunity (90.5%). Correct responses were lower for HMO presence in infant formula (39.7%) and resistance to stomach acidity (51.9%), indicating specific perception gaps. Figure 3 shows that perception scores were mainly clustered in the moderate-to-high range, with mean perception score of 5.1 (SD = 1.25). No sociodemographic variable showed a statistically significant difference in perception score. Cronbach’s α was 0.318, indicating poor internal consistency. This finding was expected because the seven items intentionally assessed different domains of HMO perception rather than a single underlying construct. Accordingly, the perception score should be interpreted as an exploratory descriptive index rather than as a validated psychometric scale.

**Table 2:** Future interest, recommendations, formula preference, and information sources among parents.

Section	Item/Response	*n* (%)
Future interest	Should HMO-enriched formula exist and be used in Qatar? – Strongly agree	57 (30.2%)
	Agree	64 (33.9%)
	Neutral	61 (32.3%)
	Disagree	2 (1.1%)
	Strongly disagree	5 (2.6%)
Recommendation	Would recommend to a mother who cannot breastfeed exclusively – Yes	82 (43.4%)
	Not sure	94 (49.7%)
	No	13 (6.9%)
Recommendation by HCP	Are HMOs enriched formulas recommended by healthcare providers in Qatar-yes	22 (11.6%)
	Not sure	121 (64.0%)
	No	46 (24.3%)
Interest to know	Would be interested in knowing more about HMOs – Yes	172 (91.0%)
	No	17 (9.0%)
Preference	Preferred formula if needed – HMO-enriched formula	68 (36.0%)
	Regular formula	31 (16.4%)
	No specific preference	90 (47.6%)
Effect of HMOs presence in formula on formula choice	I am not sure	88 (46.6%)
	Yes, I will be more likely to choose it	65 (34.4%)
	No, it will not affect my choice	36 (19.0%)
Information sources	Healthcare provider	69 (36.5%)
	Social media/internet	51 (27.0%)
	Scientific literature	50 (26.5%)
	Friends and family	8 (4.2%)
	Formula packaging	8 (4.2%)
	I do not prefer infant formula	1 (0.5%)
	I would prefer multiple sources because one source is not enough	1 (0.5%)
	No preference	1 (0.5%)

Item-specific percentages are based on non-missing responses. The most preferred information source was HCPs.

**Figure 3:**
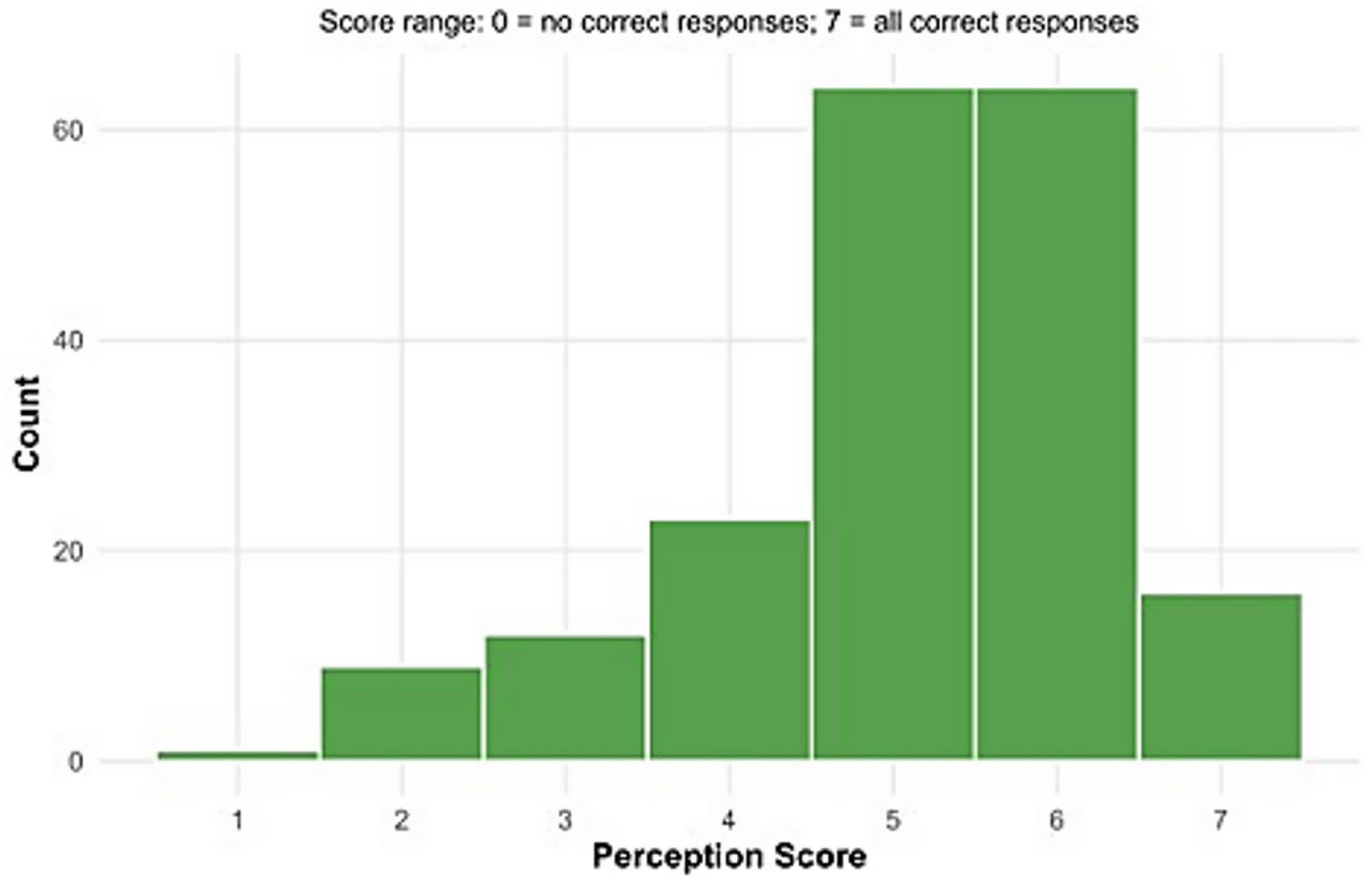
Distribution of parent perception scores (0–7), *n* = 189. Mean = 5.1, SD = 1.25, median = 5.

#### 3.1.2 HCPs survey results

A total of 64 HCPs responses were collected. Of these, 61 provided consent and were retained for analysis, while 3 HCPs (4.7%) did not consent and were excluded. Among retained responses, most respondents were female (67.2%), aged 41–50 years (36.1%) and had more than 10 years of experience (68.9%), with pediatricians forming the largest professional group (41%). The vast majority worked in government hospitals (96.7%). The number of infant patients managed varied, with a considerable proportion reporting moderate-to-high infant exposure, suggesting active clinical involvement. The demographic and professional characteristics of participating HCPs, along with a summary of their responses to the perception items, are shown in Table 3.

**Table 3:** Demographic and professional characteristics of HCPs.

Characteristic	Category	*n* (%)
Gender	Female	41 (67.2%)
	Male	20 (32.8%)
Age group	25–30	10 (16.4%)
	31–40	17 (27.9%)
	41–50	22 (36.1%)
	51–60	9 (14.8%)
	>50	3 (4.9%)
Professional role	Pediatrician	25 (41%)
	Dietitian	16 (26.2%)
	Nurse	15 (24.6%)
	Breastfeeding consultant	1 (1.6%)
	Breastfeeding specialist	1 (1.6%)
	Lactation consultant	1 (1.6%)
	Lactation specialist	1 (1.6%)
	Midwife and Lactation consultant	1 (1.6%)
Years of experience	1–4 years	10 (16.4%)
	5–10 years	9 (14.8%)
	>10 years	42 (68.9%)
Infant patient number	<10%	21.3%
	10–25%	16.4%
	26–50%	29.5%
	>50%	32.8%
Work setting	Governmental hospital	59 (96.7%)
	Specialized clinic	1 (1.6%)
	I work alone from my home	1 (1.6%)
HCP perception item answered correctly	Natural components in human milk	56 (91.8%)
	Available in infant formulas	42 (68.9%)
	Destroyed by stomach acidity (correct = no)	18 (29.5%)
	Important for intestinal flora	57 (93.4%)
	Important for immunity	58 (95.1%)
	Reduces allergic reactions.	57 (93.4%)
	Negative brain effect (correct = no)	18 (29.5%)

Perception of HMOs among HCPs was generally moderate to high. A high proportion of participants correctly identified HMOs as natural components of human milk (91.8%), their role in intestinal flora (93.4%), immunity (95.1%), and the reduction of allergic responses (93.4%). Perception gaps were observed for resistance to stomach acidity (29.5%) and presence in infant formulas (68.9%). The perception score distribution is shown in Figure 4, with mean of 5.02 (SD = 1.02). Scale reliability was low (Cronbach’s α was 0.178), reflecting low internal consistency across the heterogeneous knowledge items. The score was therefore interpreted only as an exploratory descriptive index summarizing factual HMO knowledge rather than as a validated measurement scale.

**Figure 4:**
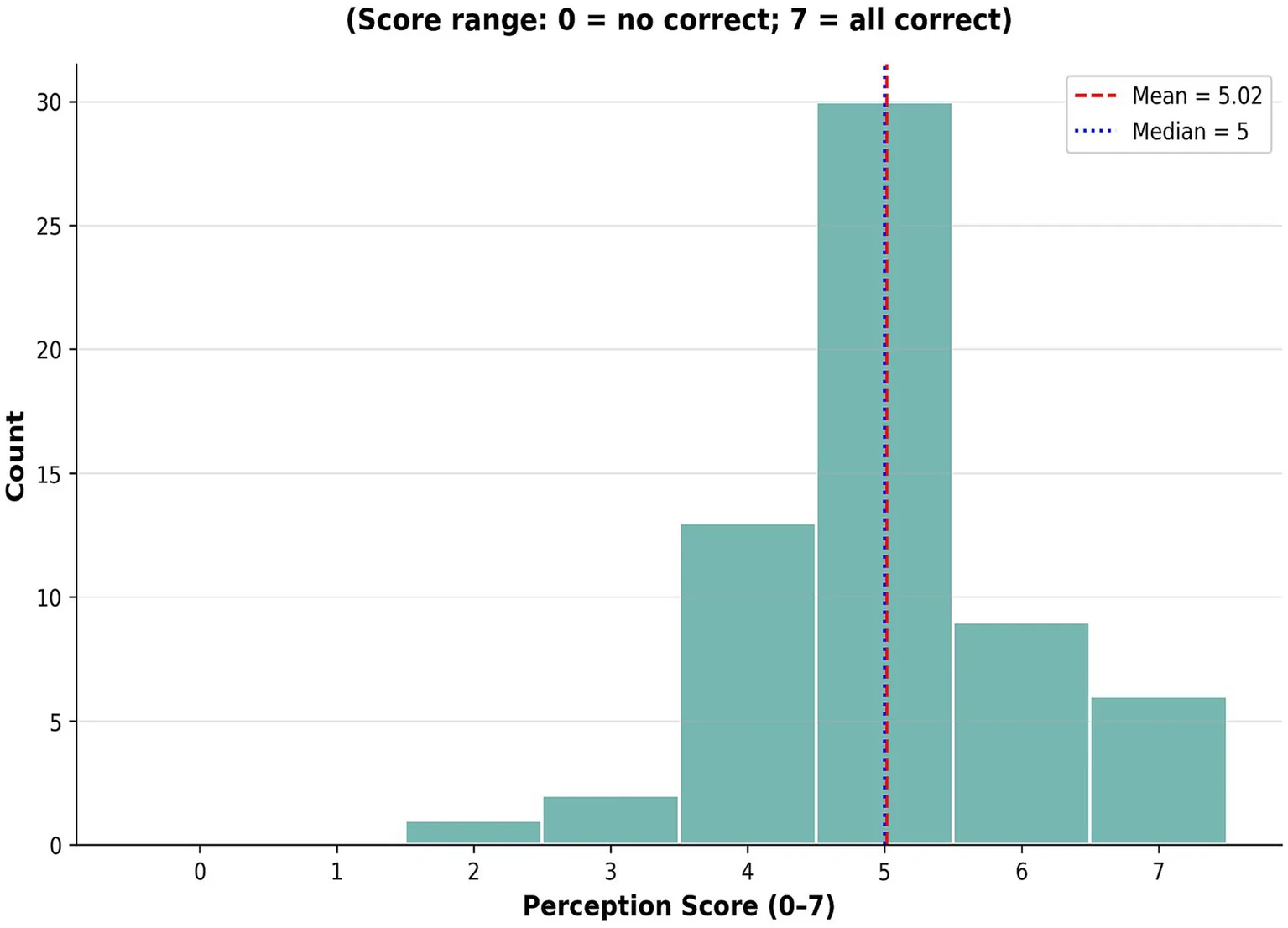
Distribution of HCP perception scores (0–7), *n* = 61. Mean = 5.02, *SD* = 1.02, Median = 5. HCPs. The perception score ranged from 0 to 7, where 0 indicates no correct responses and 7 indicates all responses correct.

Table 4 shows that the majority of HCPs heard about HMOs (75.4%) and an HMO-enriched formula (67.2%), indicating relatively high familiarity with the concept. However, self-rated perception was mostly fair, with nearly half of participants reporting Fair perception (47.5%), suggesting variability in depth of understanding. Formal training was limited, with only 13.1% of HCPs reporting having received training. Regarding public perception of HMOs in Qatar, 50.8% believed it was low, and 34.4% were uncertain. Regarding willingness to use HMO-enriched formula in Qatar, 55.7% of HCPs were “not sure.”

**Table 4:** Perception, willingness to use, training HCPs clinical practice and future interest of HCPs.

Characteristic	Category	*n* (%)
Heard about HMOs	Yes	46 (75.4%)
	No	15 (24.6%)
Self-rated perception of HMOs	Poor perception	18 (29.5%)
	Fair perception	29 (47.5%)
	Good perception	14 (22.9%)
Heard about HMO-enriched formula	Yes	41 (67.21%)
	No	20 (32.7%)
Formal training on HMOs	Yes	8 (13.1%)
	No	53 (86.8%)
Adequate awareness about HMOs in Qatar	Yes	9 (14.7%)
	No	31 (50.8%)
	Not sure	21 (34.4)
Willingness to use HMO-enriched formula in Qatar	Yes	14 (23%)
	No	13 (21.3%)
	Not sure	34 (55.7%)
Ever advised HMO-enriched formula	Yes	25 (41%)
	No	36 (59%)
If yes advised, then how do you recommend HMOs enriched formula to parents	Sometimes	15 (35.7%)
	Never	11 (26.3%)
	Often	9 (21.4%)
	Always	4 (9.5%)
	Rarely	3 (7.1%)
Interested in learning more about HMOs	Yes	52 (81.2%)
	Not sure	5 (7.8%)
	No	4 (6.2%)
Recommendations to mothers	Yes	50 (82%)
	Not sure	10 (16.4%)
	No	1 (1.6%)

A total of 41% of HCPs reported having advised HMO-enriched formula, indicating that clinical recommendation was not yet widely established. Among those who provided advice, recommendations were generally inconsistent and infrequent, with many reporting that they either do not recommend it regularly or do so only occasionally, reflecting variability in clinical practice. Despite this, substantial interest in further education, with 81.2% of HCPs expressing willingness to learn, indicated strong willingness to receive additional training and information. Furthermore, most HCPs (82%) indicated that they would recommend an HMO-enriched formula to mothers who cannot breastfeed exclusively, suggesting generally positive views toward its potential use when appropriate.

Boxplots illustrate the distribution of perception scores among HCPs who have heard about HMOs versus those who have not. The median perception score appears slightly higher among those who have heard of HMOs, but the difference was not statistically significant (*p* = 0.111). Greater variability was observed in the group who have heard of HMOs (Figure 5).

**Figure 5:**
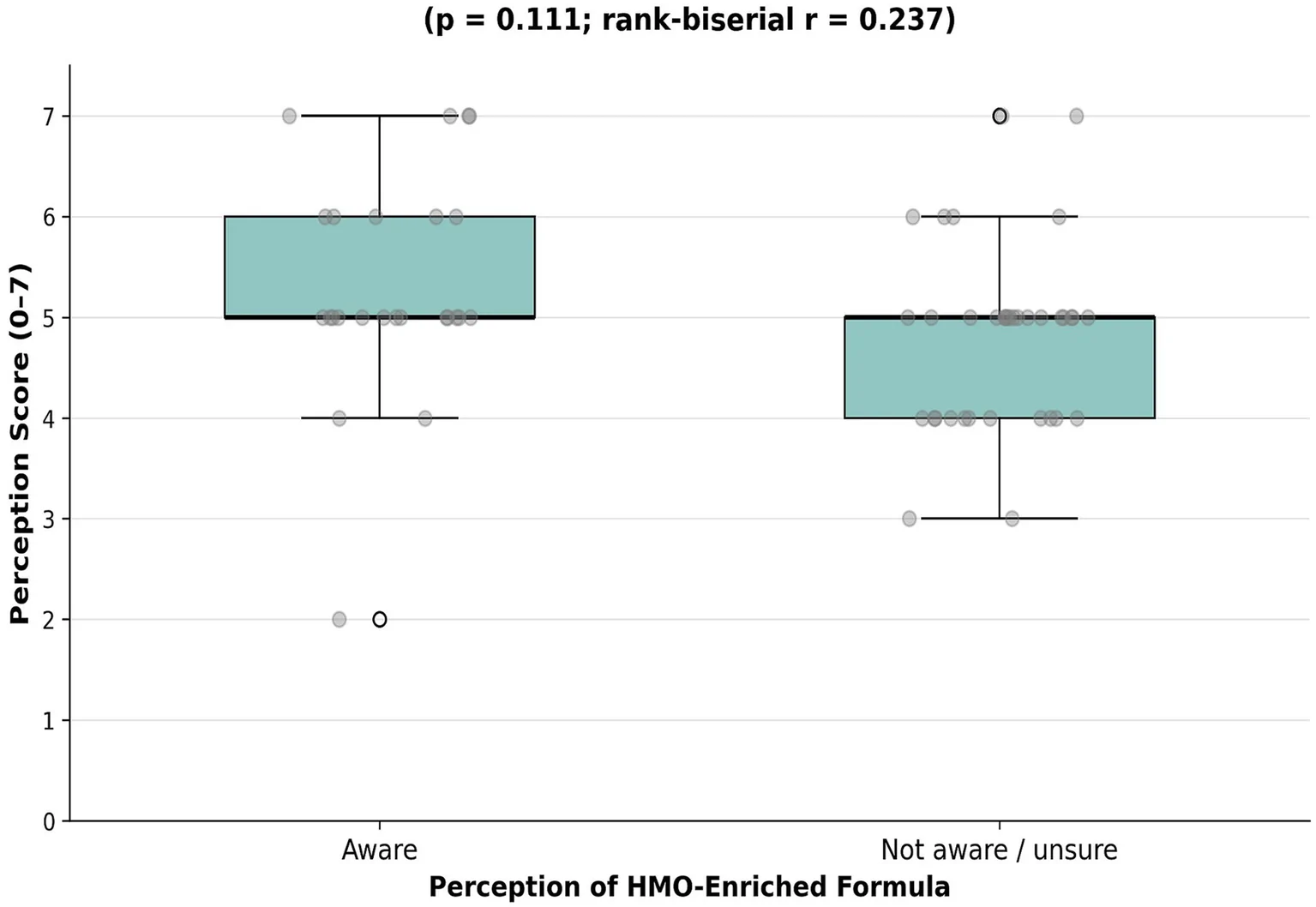
HCP perception scores by having heard of HMO-enriched formula (*n* = 61). No significant difference (Mann–Whitney U, *p* = 0.111; rank-biserial *r* = 0.237).

HCPs identified 4 main barriers that may limit parental choice of HMO-enriched formula. The most frequently reported barrier was limited HMOs perception, followed by high cost and limited availability, concerns about safety, and cultural factors. These barriers may suggest that uptake may be related not only to product access, but also to clear evidence-based communication and improved parent education.

#### 3.1.3 Inferential analysis

The parent perception score failed the Shapiro–Wilk normality test (W = 0.887, *p* < 0.001), and the HCP perception score also failed the normality test (W = 0.89, *p* < 0.001). This means the score distribution was not normal in either dataset. The F test of variance was non-significant in both the parent dataset (*p* = 0.536) and the HCP dataset (*p* = 0.600), indicating no strong evidence of variance inequality between the compared groups. Because the normality assumption was violated, non-parametric tests were preferred for score comparisons: Mann–Whitney U for two-group comparisons and Kruskal-Wallis for three-group comparisons. To account for multiple hypothesis testing, FDR adjustment was applied to the relevant inferential analyses. Tables 5, 6 summarize the statistical analysis and the *p*-values for each test used.

**Table 5:** Parent inferential analysis.

Analysis	Statistic	*p* value	FDR-adjusted *p*-value	Effect size	Effect size type
Shapiro–Wilk normality: perception score	0.887	*p* < 0.001	<0.001		
Mann–Whitney: perception score by HMO awareness	2347.5	0.151	0.227	0.148	Rank-biserial r
Mann–Whitney: perception score by HMO-formula awareness	1849	0.014	0.042	0.264	Rank-biserial r
Kruskal-Wallis: perception score by willingness to use	2.752	0.252	0.252	0.004	Epsilon-squared
Chi-square/Fisher: nationality vs HMO-formula awareness	5.496	0.028	0.060	0.171	Cramér’s V
Chi-square/Fisher: HMO-formula awareness vs willingness to use	6.421	0.040	0.060	0.184	Cramér’s V
Chi-square/Fisher: education vs willingness to use	7.741	0.459	0.459	0.143	Cramér’s V
Spearman: perception score vs HMO-formula awareness	0.202	0.010	0.030		
Spearman: HMO-formula awareness vs willingness to use	0.164	0.037	0.056		
Spearman: perception score vs willingness to use	0.076	0.295	0.295		

**Table 6:** HCP inferential analysis.

Analysis	Statistic	*p* value	FDR-adjusted *p*-value	Effect size	Effect size type
Shapiro–Wilk normality: perception score	0.89	*p* < 0.001	<0.001		
Mann–Whitney: perception score by HMO-formula awareness	313	0.111	0.111	0.237	Rank-biserial r
Chi-square/Fisher: HMO-formula awareness vs advised formula	15.93	*p* < 0.001	0.002	0.511	Cramér’s V
Chi-square/Fisher: professional role vs HMO-formula awareness	13.213	0.067	0.067	0.465	Cramér’s V
Spearman: perception score vs HMO-formula awareness	0.207	0.110	0.110		
Spearman: perception score vs advised formula	0.294	0.021	0.031		
Spearman: HMO-formula awareness vs advised formula	0.511	*p* < 0.001	0.003		

Among parents, nationality showed a nominal association with awareness of HMO-enriched formula before adjustment (χ^2^ = 5.496, unadjusted *p* = 0.028; Cramér’s V = 0.171), with awareness being higher among non-Qatari parents. However, this association did not remain statistically significant after FDR adjustment (pFDR = 0.060). Age group, education level, and number of children were not significantly associated with perception, and education level was not significantly associated with willingness to use HMO-enriched formula (*p* = 0.459; pFDR = 0.459).

The perception score did not differ significantly between parents who had or had not heard about HMOs (Mann–Whitney U = 2347.5, *p* = 0.151; pFDR = 0.227). In contrast, perception scores differed significantly according to HMO-enriched formula awareness (Mann–Whitney U = 1849, *p* = 0.014; pFDR = 0.042), with a small-to-moderate effect size (rank-biserial r = 0.264). Spearman correlation similarly demonstrated a significant positive association between perception score and HMO-enriched formula awareness (ρ = 0.202, *p* = 0.010; pFDR = 0.030).

The association between HMO-enriched formula awareness and willingness to use the formula was nominally significant before multiple-testing adjustment (*p* = 0.040; Cramér’s V = 0.184), but it did not remain statistically significant after FDR correction (pFDR = 0.060). Similarly, the Spearman correlation between HMO-enriched formula awareness and willingness to use was nominally significant before adjustment (ρ = 0.164, *p* = 0.037) but not after FDR correction (pFDR = 0.056). Perception score was not significantly associated with willingness to use HMO-enriched formula (ρ = 0.076, *p* = 0.295; pFDR = 0.295), consistent with the logistic regression findings.

Among HCPs, HMO-enriched formula awareness was strongly associated with having advised HMO-enriched formula to parents, and this association remained statistically significant after FDR adjustment (χ^2^ = 15.930, *p* < 0.001; pFDR = 0.002; Cramér’s V = 0.511). Professional role was not significantly associated with HMO-enriched formula awareness (*p* = 0.067; pFDR = 0.067). Perception scores did not differ significantly between HCPs who had and had not heard of HMO-enriched formula (Mann–Whitney U = 313, *p* = 0.111; pFDR = 0.111).

Spearman correlation further demonstrated that awareness of HMO-enriched formula was positively associated with advising behavior, and this association remained statistically significant after FDR adjustment (ρ = 0.511, *p* < 0.001; pFDR = 0.003). Perception score was also positively associated with advising behavior (ρ = 0.294, *p* = 0.021; pFDR = 0.032), whereas its association with HMO-enriched formula awareness was not statistically significant (ρ = 0.207, *p* = 0.110; pFDR = 0.110). All reported associations are observational and should not be interpreted as causal.

#### 3.1.4 Subgroup analysis by nationality

To examine whether the primary study outcomes differed according to nationality, an additional subgroup analysis compared Qatari and non-Qatari parents. The four primary outcomes are summarized in Table 7, while additional nationality-based comparisons are provided in Supplementary Table 1. Benjamini-Hochberg FDR adjustment was applied across the 13 nationality comparisons.

**Table 7:** Comparison of primary survey outcomes between Qatari and non-Qatari parents.

Outcome	Qatari	Non-Qatari	Test	Unadjusted p	FDR-adjusted p	Effect size
HMO awareness	17.4% aware	23.5% aware	Fisher exact	0.404	0.540	V = 0.069
HMO-enriched formula awareness	13.0% aware	27.5% aware	Fisher exact	0.028	0.180	V = 0.171
Perception score	5.0 (5.0–6.0)	5.0 (5.0–6.0)	Mann–Whitney U	0.576	0.680	r = 0.051
Willingness to use HMO-enriched formula: Yes/Possibly/No	16.7%/62.3%/21.0%	23.5%/62.7%/13.7%	χ^2^	0.370	0.540	V = 0.103

Awareness of HMOs did not differ significantly between Qatari and non-Qatari parents (17.4% vs. 23.5%; Fisher’s exact *p* = 0.404, pFDR = 0.540; Cramér’s V = 0.069). Awareness of HMO-enriched formula was higher among non-Qatari than Qatari parents (27.5% vs. 13.0%) and showed a nominal association before adjustment (Fisher’s exact *p* = 0.028; Cramér’s V = 0.171); however, this association did not remain statistically significant after FDR correction (pFDR = 0.180).

Perception scores were similar between groups, with both Qatari and non-Qatari parents having a median score of 5.0 (IQR: 5.0–6.0; Mann–Whitney U = 3339.5, *p* = 0.576, pFDR = 0.680; rank-biserial r = 0.051). Willingness to use HMO-enriched formula was also comparable across nationality groups (χ^2^ = 1.987, *p* = 0.370, pFDR = 0.540; Cramér’s V = 0.103).

Across the additional outcomes, reported HCP recommendation of HMO-enriched formula showed the largest observed nationality-related effect (Cramér’s V = 0.238) and was nominally significant before multiple-testing adjustment (*p* = 0.005), but did not remain statistically significant after FDR correction (pFDR = 0.060). No other nationality-based comparison was statistically significant after FDR adjustment. Hence, the evaluated awareness, perception, willingness, and related formula-use outcomes were broadly comparable between Qatari and non-Qatari parents after accounting for multiple testing.

### 3.2 Qualitative results

#### 3.2.1 Parents’ perspectives

Nine mothers participated in two FG discussions. FG1 included four mothers, and FG2 included five mothers. All participants were mothers of infants or young children under two years of age residing in Qatar.

Using thematic analysis, four themes were inductively generated from the discussions. These themes captured mothers’ infant feeding experiences, factors influencing infant formula selection, limited prior familiarity with human milk oligosaccharides (HMOs), and cautious willingness to consider HMO-enriched formula when clear evidence of safety and benefit is available. The generated themes, subthemes, key findings, and representative quotes are presented in Table 8.

**Table 8:** Themes and subthemes generated from the parents’ FG discussions.

Theme	Key findings	Supporting quotes
Theme 1. Infant feeding experiences and perceived challenges		
Sub-theme 1.1: The primacy of breastfeeding and barriers to sustaining it	Most mothers preferred breastfeeding because they associated it with immune protection, emotional bonding, and confidence in the safety of infant nutrition. However, sustaining breastfeeding was often difficult, mainly because of insufficient milk supply, illness, physical discomfort, and the need to store milk.	“The challenge is how to keep the milk flowing and ensure it meets the baby’s needs.” (Speaker 1) “How to maintain the milk supply in a sufficient amount or make a stored supply if I am not present.” (Speaker 2) “The second one, around one month I breastfed her naturally, I had fever, that fever, this one that made me go down, and I used to pump. After a month, I got blood poisoning. So, I was forced to stop breastfeeding her and introduce the formula milk.” (Speaker 3)
Sub-theme 1.2: Circumstances leading to formula introduction	Most mothers described formula introduction as a practical response to specific circumstances rather than as their initial feeding preference. Common triggers included early separation from the infant after birth, insufficient milk supply, infant bottle preference, and returning to work or university.	“She got used to formula, and after that I tried to breastfeed her, but she would not accept except with the bottle.” (Speaker 3) “The main difficulties are physical pain and trying to store milk because I will return to university.” (Speaker 9) “Formula is only coverage during work hours, not a healthy alternative.” (Speaker 1)
Theme 2. Factors shaping infant formula selection		
Sub-theme 2.1: Nutritional proximity to breast milk and digestive tolerance	The main criterion used by mothers when selecting formula was its perceived similarity to breast milk in composition and function. Mothers also considered key nutrients, digestive tolerance, infant acceptance, and perceived suitability for the baby’s growth.	“The most important thing I look for is something as close as possible to breast milk; I know nothing can match it, but that is what I am looking for.” (Speaker 2) “I look for components similar to breast milk and the same vitamins. I want to make sure it has the DHA, iron, and the proteins that a growing baby needs, because I feel that if it does not have these, I am not giving my baby what she truly needs.” (Speaker 1) “I gave him natural milk for five months, then due to work it decreased for me and I had to introduce formula, and I gave goat milk because they say it’s the closest milk to breast milk. I gave goat milk” (Speaker 6)
Sub-theme 2.2 Role of medical guidance, social influence, and personal experience	In addition to nutritional composition, mothers relied on healthcare advice, family and peer experiences, social observation, and their own reading. These sources helped them feel more confident when choosing or changing formula.	“I saw their children with allergies and eczema, so I said let me choose something different.” (Speaker 3) “I relied on the experiences of those around me and some reading. I would ask mothers in my family and close friends what they used and whether it worked for their babies, and then I would also look it up to see if what they said matched what the research showed.” (Speaker 8)
Theme 3. Limited perception but openness to learning about HMOs		
Sub-theme 3.1: Limited prior awareness of HMOs	Most mothers had not heard of HMOs before the FGdiscussion. Their baseline familiarity with the term, its function, and its possible presence in infant formula was limited.	“I have no idea- this is the first time I have heard about it.” (Speaker 1) “Even me, I did not know about this.” (Speaker 3) “I have no idea about them.” (Speaker 5) “I had not heard about them before except in this discussion.” (Speaker 8)
Sub-theme 3.2: Engagement with HMOs information during discussion	After receiving a brief explanation of HMOs and their incorporation into formula, mothers engaged with the topic and linked it to their existing priority: choosing formula that is closer to breast milk. Several mothers expressed interest in receiving more education and evidence on this topic.	The most important thing is that it will be more similar to breast milk.” (Speaker 3) “I would like you to educate people more about this topic and the components found in breast milk, and to publish studies tested on children. A substance with this importance, we must be educated about it, because we are hearing about it for the first time.” (Speaker 1)
Theme 4. Perceptions of and willingness to use HMO-enriched formula		
Sub-theme 4.1: Safety concerns and barriers to acceptance	Mothers’ main concern was the safety of industrially replicated HMOs and formula manufacturing. Concerns included uncertainty about formulation accuracy, recalls, preservatives, digestive symptoms, reflux, constipation, and the potential effects of unfamiliar ingredients.	“It is an imitated substance, not the same sugar in breast milk. I do not know what guarantees me that they have put the correct formulation.” (Speaker 1) “Many times, they say that formula has been recalled because it contained a toxic substance or there was a manufacturing error, and after that I fear it will cause symptoms like constipation, reflux, or being heavy on the baby’s stomach.” (Speaker 1) “The most thing that scares me in formula is preservatives. I always check the ingredients list, and when I see things I cannot pronounce or understand, I worry about what they might do to my baby over time, especially to his stomach and his liver.” (Speaker 3) One of the participants said that she would prefer less processed versions because she believed that formula is the default choice when fresh goat milk, when pasteurized properly, could serve as a better alternative (Speaker 4).
Sub-theme 4.2: Conditions and perceived benefits that may facilitate use	Most mothers did not reject HMO-enriched formula. However, they indicated that they would consider it only if it was supported by reliable studies, visible benefits, positive user experience, and recommendations from trusted healthcare professionals.	“This idea itself encourages me; they are trying to put something from breast milk into formula to make it closer.” (Speaker 2) “I will not be the first to try it. I will wait and see more than one person who has tried it, see its benefit on the baby whether it caused constipation, helped growth, and whether the baby accepted it. When I see more than one study or more than one experience, I might accept using it.” (Speaker 1) “Reliable studies and trials, not just marketing.” (Speaker 8) “I would use it only if recommended by a trusted doctor. Not just any doctor, but one who knows my baby and who I trust, because I need someone who has seen the evidence and is confident enough to tell me it is safe for my child.” (Speaker 7)

##### 3.2.1.1 Theme 1. Infant feeding experiences and perceived challenges

This theme describes mothers’ experiences with infant feeding and the challenges they faced. Breastfeeding was generally viewed as the preferred form of infant nutrition. However, sustaining breastfeeding was often affected by physiological, practical, and situational factors, including illness, milk supply, infant acceptance, and return to work or education.

##### 3.2.1.2 Theme 2. Factors shaping infant formula selection

This theme explores the criteria mothers used when selecting infant formula. The most common considerations included nutritional composition, similarity to breast milk, digestive tolerance, infant acceptance, advice from healthcare professionals, and the experiences of family members and peers.

##### 3.2.1.3 Theme 3. Limited awareness but openness to learning about HMOs

This theme explores mothers’ prior familiarity with HMOs and their level of engagement with the topic during the discussion. Although most participants had little or no prior awareness of HMOs, they were able to engage meaningfully with the concept after introductory information was provided.

##### 3.2.1.4 Theme 4. Cautious willingness to use HMO-enriched formula under safety and evidence conditions

This theme captures mothers’ attitudes toward HMO-enriched formula, their concerns, and the conditions under which they may consider using such products. Mothers expressed willingness in principle, but this willingness depended on safety assurance, reliable evidence, and trusted medical guidance.

#### 3.2.2 HCPs’ perspectives

Twelve HCPs from different professional backgrounds participated in three FG discussions. FG1 included five HCPs, FG2 included four, and FG3 included three.

Participants included four dietitians, two breastfeeding consultants, two breastfeeding specialists, two pediatric physicians, and two midwives or nurses. The discussions explored healthcare providers’ perceptions and clinical practices related to infant feeding, HMOs, and HMO-enriched infant formula.

Using thematic analysis, five themes were generated to categorize participants’ insights. These addressed the importance of breastfeeding in infant nutrition, perceptions of HMOs, use of HMO-enriched formula, challenges in recommending HMO-enriched formula, and willingness to recommend HMO-enriched formula. The generated themes, subthemes, key findings, and representative quotes are presented in Table 9.

**Table 9:** Themes and subthemes generated from the HCPs FGs.

Theme	Key findings	Supporting quotes
Theme 1: Importance of breastfeeding in infant Nutrition		
Sub-theme 1.1: Breastfeeding as the optimal nutrition	Participants emphasized breastfeeding as the first choice feeding option because it provides essential nutrients, supports infant development, is easily available, and is generally easy to digest.	“Breastfeeding is the best nutrition for the baby’s health and mother’s health also. we are supporting, encouraging and protecting it to the last moment.” (Speaker 1, Breastfeeding Specialist) “First choice always for the exclusive breastfeeding, because it is easily available, easy to digest, and it’s overall development of the baby from head to foot and the brain development.” (Speaker 5, Nurse) “First choice always for the breast milk… because it is easily available and easy to digest.” (Speaker 7, NICU Dietitian) “Yes, so we always recommend, and we always mention to mothers and parents that mother’s milk is the best nutrition for the babies, because it provides the best nutrition and benefit to the babies, and there’s no superior milk to the mother’s milk.” (Speaker 11, Pediatric Resident)
Sub-theme 1.2: Breastfeeding and infant immunity	Participants stressed that breastfeeding provides immune-related benefits, including protection against infections and support for gastrointestinal and respiratory health.	“What we prioritize is to start with mother’s breast milk, so that you know they will be able to benefit not just from the nutrition but also from the immune properties of the breast milk.” (Speaker 4, Breastfeeding Specialist) “This protection is vital for preventing long-term respiratory and gastrointestinal complications.” (Speaker 11, Pediatric Resident)
Sub-theme 1.3: Human milk fortification	Participants explained that human milk fortifiers are used, particularly for premature or sick infants, to increase calories, proteins, and fats and to compensate for increased nutritional requirements.	“We add human milk fortifier to increase calories, proteins and fats for premature babies.” (Speaker 4, Breastfeeding Specialist) “Because premature babies still do not have stores in their bodies for these components, and the energy expenditure is usually higher for babies who are sick or in the ICU. That’s why we have to replace or add something to the mother milk. To give them the needed to compensate the energy they lose because of sickness.” (Speaker1, Breastfeeding Specialist)
Theme 2: Perceptions of HMOs		
Sub-theme 2.1: Perceptions gaps levels	Many healthcare providers reported limited knowledge of HMOs. Some had general awareness that HMOs are components of human milk, but detailed knowledge varied across professional groups.	“I have an idea about oligosaccharide. Because I’m a breastfeeding specialist, I have seen in a course that we prepared for an exam one module about infant nutrition. So, it included very little information about oligosaccharides.” (Speaker 1, Breastfeeding Specialist) “For me, honestly, I do not know anything about it. However, I know that it’s one of the components of the breast milk” (Speaker 4, Breastfeeding Specialist) “Honestly, I have just now received the knowledge from all of them. I think it’s related to breast milk or something.” (Speaker 5, Nurse) “Most of the questions of the survey about HMOs I answered “I do not know”… I do not have that level of knowledge.” (Speaker 2, Breastfeeding Specialist) “I have general information. This is the carb— the main carb—in the mother’s milk and so it’s like main carb in the other formulas” (speaker 9, NICU Dietitian) “We, truly speaking, do not know much about it, to be honest. We only know, at least for me, that human milk has high HMOs, and colostrum has the highest. It’s important for immunity, resisting infections, maturity, and good nutrition. That’s my understanding” (Speaker 10, Pediatric Consultant)
Sub-theme 2.2: Functional understanding of HMOs role in gut health	Despite knowledge gaps, some participants described HMOs as supporting infant immunity, reducing infection risk, influencing the gut microbiome, and potentially contributing to longer-term outcomes.	“But the thing that I know that it’s important to support baby’s immune system and prevent GI tract infections and also it prevents UTI for the baby. So, oligosaccharides are attached to the epithelial cells and prevent the bacteria from attaching to these epithelial cells. In this way, it protects the baby from infections” (Speaker1, Breastfeeding Specialist) “Oligosaccharides support the baby’s immune system and prevent infections.” (Speaker 5, Nurse) “It affects the microbiome and it might increase also the immunity and infants and might go beyond that to like long-term effects later on also the Cognitive.” (Speaker 8, NICU dietitian)
Sub-theme 2.3: Limited perceptions of HMOs in Qatar’s community in HCPs’ opinion	Participants believed that awareness of HMOs is limited among both parents and healthcare providers in Qatar. Several participants also framed the issue as part of a broader global knowledge gap.	“Awareness is an issue in the clinical. This is my clinical insight” (Speaker 8, NICU Dietitian) “Lack of awareness is not only in Qatar, it’s worldwide. Because worldwide they have guideline or knowledge, definitely we will adapt those things. And we usually are updating ourselves and updating our guidelines by relying on the worldwide guidelines. If they do not have it, for sure we will not develop any separated things.” (Speaker 6, Neonate Dietitian) “Absolutely parents are not aware.” (Speaker 10, Pediatric Consultant) “So, in general, there is a lack of awareness. Worldwide. This again, why we lean more towards promoting mother’s milk, breast milk, and all which naturally contain the HMOs.” (Speaker 7, NICU Dietitian) “Even as we are working in this facility, we know we are still not confident about it. We do not have enough information about the HMOs.” (Speaker 12, Registered Midwife)
Theme 3: Use of HMO-enriched infant formula		
Sub-theme 3.1: HMO-enriched formula recommendation	Participants generally reported that they had not previously recommended HMO-enriched formula. Their recommendations focused on the overall formula profile rather than the presence of HMOs specifically.	“I will not look at oligosaccharides’ benefit; I will look at the formula itself, the whole milk. What benefits will the baby get? And what other benefits we lose if the baby gets this kind of formula, regardless of the human milk oligosaccharides. I will look at other bioactive ingredients, bioactive cells, antibodies, immune factors, and all other things that it will never be present in any other formula. So I will look at the whole meal or the whole formula the baby will get” (Speaker 2, Breastfeeding Specialist) “In general, we never focus on oligosaccharides. We never do. We have any interest in oligosaccharides of any formula” (Speaker 6, Neonate Dietitian) “No. I did not recommend it previously. We do not know that there is a formula fortified with oligosaccharides.” (Speaker 8, NICU Dietitian) “Truly speaking, we do not know much. We do not know enough to recommend.” (Speaker 10, Pediatric Consultant) “To be honest, no, not really. In general, for us, we do not recommend any specific formulas to parents. We just tell them they can get any standard formula because, for us, all formulas are the same unless there is a specific need, like cow’s milk allergy or such. For me, I’m not even aware that there are certain formulas that are enriched with human milk oligosaccharide complexes, so, no, I have not recommended it at any time.” (speaker 11, Pediatric Resident)
Sub-theme 3.2 Prioritizing other components in infant formula	When discussing infant formula, participants reported prioritizing calories, protein type, hydrolysis status, iron, vitamin D, and medical indications rather than specific carbohydrates such as HMOs.	“I will look at the protein itself, whether it is hydrolyzed or not.” (Speaker 1, Breastfeeding Specialist) “Well, the first thing my finger will go on is the calorie, next, the protein, and we never go deeper than how many calories, even not the carb, we will never count the carb, we will never count the fat, we will focus on calorie, total calorie. We will go for the protein. Rarely we go for iron, Vitamin D. Very rare for some conditions, and very rare in neonates. But for a specific carb or specific oligo or whatever, we never check that.” (Speaker 6, Neonate Dietitian)
Theme 4: Challenges in recommending HMO-enriched infant formula.		
Sub-theme 4.1: Cost and affordability	Participants identified cost as a major potential barrier. They noted that if HMO-enriched formulas are priced higher than standard formulas, some families may not be able to afford them.	“Maybe the price. If it is more costly, parents will not be able to afford it.” (Speaker 1, Breastfeeding Specialist) “I just think the financial outcome could also be a problem, because if companies are claiming that they are rich in prebiotics, they will price their formulas higher than the other formulas. And not everyone can afford that.” (Speaker 7, NICU Dietitian) “If it is more costly, parents will not be able to afford it.” (Speaker 9, NICU Dietitian) “Cost is important, as our colleague said. We have people here at different levels; some can afford it, some cannot. But if we know the benefits, then everything else falls into place. This is how I can convince families to shift from one formula to another.” (Speaker 10, Pediatric Consultant) “Maybe cost” (Speaker12, Registered Midwife)
Sub-theme 4.2: Lack of professional perceptions and market availability	Participants described limited professional awareness and uncertainty about market availability as barriers to recommending HMO-enriched formula. They emphasized the need for clearer information about benefits and product access.	“Another thing is probably the awareness that this type of thing exists. Because even when we are talking with dieticians here in the hospital, they do not recommend a formula based on the presence of HMOs. They just say it has to be 24 calories, or it must be 26. So, it’s not very specific.” (Speaker 4, Breastfeeding Specialist) “Even healthcare providers are not aware about that. awareness and availability are important.” (Speaker 3, Breastfeeding Specialist) “Maybe availability” (Speaker 12, Registered Midwife) “The knowledge about HMO, what is HMO, what does it make, and what is the benefit of taking it? What benefit will the child gain from a formula that has high HMO compared to one that has low HMO?” (Speaker 10, Pediatric Consultant)
Sub-theme 4.3: Safety concerns and tolerance	Safety, allergy risk, taste, texture, and infant tolerance were raised as possible concerns. Participants noted that synthetic or added complexes may require careful evaluation before being recommended.	“I believe that because these complexes would be synthesized, allergy is one of the things we should look for. Also, tolerance—I’m not sure how these complexes might change the taste and texture of the formula. Because certain formulas do not necessarily have a good taste or smell, and many babies suffer because they do not tolerate it. So, I’m not sure, if these formulas are enriched with these complexes, how the taste and texture would be different.” (Speaker 11, Pediatric Resident)
Theme 5: Willingness to recommend HMO-enriched formula		
Sub-theme 5.1: Irreplaceability of breastfeeding	Most participants emphasized that HMO-enriched formula cannot replace breastfeeding. They viewed human milk as biologically unique and argued that fortification alone cannot reproduce the full benefits of breastfeeding.	“Even if it is fortified or oligosaccharide is added, bioavailability of oligosaccharide will be much less than the amount in mother’s milk.” (speaker 1, Breastfeeding Specialist) “Mother’s milk is designed according to the baby’s requirements and cannot be substituted.” (Speaker 3, Breastfeeding Consultant) “Even if it is fortified or oligosaccharide is added, bioavailability of oligosaccharide will be much less than the amount in mother’s milk.” (Speaker 1, Breastfeeding Specialist)
Sub-theme 5.2: Perceived benefits of HMO-enriched formula	Some participants indicated that adding HMOs to formula could be beneficial for infants who cannot be breastfed, provided that the formulation is safe, evidence-based, and used in appropriate proportions	“If synthesized HMOs are added to baby formula, it will definitely benefit, as long as it is a good percentage relative to human milk, it’s definitely beneficial. But we need to know more, we need to orient ourselves more before we can recommend it to parents and families. We recommend human milk because we know it has everything that is good. But for other formulas to be enriched with HMO, we have to dig deeper, and we have to be aware ourselves before we can give awareness to parents and families… If we know the benefits of HMOs, then everything else falls into place. This is how I can convince families to shift from one formula to another.” (Speaker 10, Pediatric Consultant) “So again, because I do not know much about human milk oligosaccharides, but we know for sure if it’s something that is available in mother’s milk and breast milk in a good proportion, we believe it’s a good thing and has good benefits for the baby. Of course, as Speaker 12 mentioned, because not all babies are able to be breastfed, providing these important complexes in the formula would be a good thing, taking into consideration that they are done in a safe way and have no negative effect or harm to the baby.” (Speaker 11, Pediatric Resident) “If it is there, it is artificial, but it is still there. It is good because some babies cannot drink from the mother. They have some contraindications to breastfeeding. So, if there is a replacement in the formula like this HMO—as I told before, if it is near to human milk, it is really good. It has real benefits. So actually, it’s needed because we still need a formula. So, if it is rich with HMO, of course, perfect.” [Speaker 12, Registered Midwife]

##### 3.2.2.1 Theme 1. Importance of breastfeeding in infant nutrition

This theme reflects the strong emphasis placed by HCPs on breastfeeding as the optimal feeding option for infants. Participants highlighted its availability, digestibility, nutritional value, role in growth and development, and immune-related benefits. Human milk fortification was discussed mainly in relation to premature or medically vulnerable infants.

##### 3.2.2.2 Theme 2. Perceptions of HMOs

This theme explores HCPs’ familiarity with HMOs. Participants showed variable and often limited awareness. Some participants recognized HMOs as components of human milk and linked them to gut health and immunity, while others reported limited knowledge and a need for further professional education.

##### 3.2.2.3 Theme 3. Use of HMO-enriched infant formula

This theme examines whether HCPs were aware of HMO-enriched formulas and whether they recommended them to parents. Most participants reported that they had not recommended HMO-enriched formula and that their clinical focus usually remained on the overall formula type, calories, protein profile, and medical indications.

##### 3.2.2.4 Theme 4. Challenges in recommending HMO-enriched infant formula

This theme identifies potential barriers to recommending HMO-enriched formula. Participants discussed cost, affordability, market availability, limited professional awareness, lack of clear guidance, safety concerns, allergy risk, taste, texture, and infant tolerance.

##### 3.2.2.5 Theme 5. Willingness to recommend HMO-enriched formula

This theme captures HCPs’ willingness to consider or recommend an HMO-enriched formula. Participants generally emphasized that breastfeeding cannot be replaced. However, some considered HMO-enriched formula potentially beneficial for infants who cannot be breastfed, provided that safety, evidence, and appropriate clinical guidance are available.

### 3.3 Data integration

Supplementary Table 2 presents the integration of findings from the quantitative and qualitative phases. This Joint Display Table links each quantitative domain and survey item to its corresponding qualitative theme, subtheme, and an illustrative participant quote, organized into three section: Aligning results, Expanding results, and Contrasting results, thereby showing where the survey and FG data converge, where the qualitative data add explanatory depth, and where the two data sources diverge, as previously recommended for mixed-methods integration (44). Across parents and HCPs, the integrated findings showed limited baseline awareness of HMOs and HMO-enriched formula, but strong interest in learning more and conditional willingness to use or recommend these products. Among parents, the quantitative findings showed low prior awareness but high receptiveness to further information, while the qualitative findings explained that mothers’ willingness depended on safety assurance, clear evidence, and trusted medical guidance. Among HCPs, the quantitative findings showed higher awareness than parents but limited formal training and inconsistent recommendation practices, while the qualitative findings clarified the need for professional education, clear clinical guidance, and stronger evidence before routine counseling. Overall, the integrated findings suggest that the future uptake of HMO-enriched formula in Qatar may depend on HCP-led communication, structured education, safety reassurance, affordability, availability, and evidence-based guidance.

## 4 Discussion

To our knowledge, this study is the first in Qatar and perhaps in the larger Arab Gulf region to showcase exploratory perceptions of HMOs and willingness to use HMO-enriched infant formula among two distinct groups: parents of young children and HCPs specialized in infant care. Due to the increasing global interest in HMOs as well as their addition to infant formula to approximate human milk (24), understanding perceptions toward HMOs and the use of HMO- enriched formula is important to support developing evidence-based recommendations regarding infant nutrition. The current study contributes to baseline, overall representation of such knowledge using an integrated mixed-method design. It should be acknowledged, however, that the study findings should be interpreted cautiously as the HMO perception score was developed as an exploratory knowledge summary rather than a psychometrically validated instrument. The relatively low internal consistency of this exploratory index reflects the intentionally heterogeneous nature of the items, each addressing different biological and clinical aspects of HMOs, and therefore does not necessarily imply that the individual items lack informational value.

Overall, the findings of the study showed contrast between parents and HCPs regarding perception. Whereas parents had limited encounters of HMOs and HMO-enriched formula, HCPs showed comparatively more exposure. Although parents were correct in their recognition of some perception items, but the level of perception was relatively low on other items such as the existence of HMOs in commercially available baby food and their ability to resist stomach acid. The limited parental perception of HMOs reported here is in line with previous findings, where parental perception of specific bioactive formula ingredients was low despite the rapidly growing scientific and commercial activity in this field (45, 46). These results may indicate gaps in informational background of products, yet, not an overall lack of biological perception among parents. It is possible that parents may be prepared for intellectually comprehending HMOs; they just have not been exposed to the term or the product category in a manner that triggers their understanding. The qualitative results were consistent with this interpretation, with most mothers reporting no prior familiarity with HMOs. Nevertheless, after HMOs were introduced as naturally occurring components of breast milk that enhance immune and gastrointestinal health, participants were able to understand and respond to the concept effectively. Parental educational programs may, therefore, need to leverage the perception generalizations that parents in Qatar may have, rather than introduce fundamental science and basic information about HMOs, as highlighted by the above findings. It is worth mentioning that our survey items assessing baseline perceptions of HMOs were presented to participants before items on willingness to use HMO-enriched formula, in order to capture spontaneous, uninformed perceptions, without promotional statements or descriptions of HMO benefits, and with neutral wording in the willingness items. Consequently, willingness responses primarily reflect spontaneous judgments following a baseline perception exploration, with minimal informational prompting, rather than responses following an educational explanation. While this survey design keeps the risk of positive framing bias from benefit-introducing prompts minimal, the mere presentation of perception items may still have provided some contextual information that may positively influence the stated willingness, and should be acknowledged (47, 48). Future research should, therefore, compare uninformed willingness which is assessed before any HMOs-related items with informed willingness which is assessed after educational explanation, to quantify the effect of information prompts and framing on participant responses.

The analysis of the association between perception and willingness to use HMO-enriched formula among parents showed a nominally significant relationship before adjustment for multiple testing (*p* = 0.040), but this association did not remain statistically significant after FDR correction. Parents who had heard about HMO-enriched formula scored higher on perception items, which may indicate that exposure to HMO-related information may show higher odds of a better-developed perception level. These findings suggest that both perception and hearing about HMOs among parents do not individually determine behavioral intention, and decision-making in this area may be related to a broader set of factors, such as subjective norms, professional recommendations, or social media influences. Combined, these results may support that behavioral intention is affected not only by attitude but also by subjective norms and social exposure, as the Theory of Planned Behavior mentions (49). The results also conform with earlier findings that knowledge is not a reliable associated factor of dietary behavior, while attitudinal, social, and contextual factors are stronger factors affecting food-related choices (50). In light of our findings, positive perceptions or willingness will not necessarily translate into actual HMO-enriched formula adoption or real changes in infant nutrition practices, and different economic, regulatory, or cultural factors may be related.

A subgroup analysis comparing Qatari to non-Qatari parents did not show significant differences in perceptions of HMOs or willingness to use HMO-enriched formula. In a country like Qatar with unique demographic profile and a large expatriate population, contextual and experiential factors, including previous residence in countries with possible wider markets of HMO formulas, or access to multinational social and professional networks, may influence perception gaps. The expatriate population in the Gulf Region has diverse health product perception profiles based on their various healthcare backgrounds (51), and previous findings show that the country of origin and previous experience with the healthcare system significantly affect knowledge and information-seeking behavior of immigrants regarding health services (52). Yet, nationality and other sociodemographic factors did not show a statistically significant difference in our outcomes of perception and willingness to use HMO-enriched formula, perhaps suggesting that the main area of intervention in Qatar may be raising awareness about HMOs equally across different sectors of the population.

Moreover, FG discussions indicated the use of the formula as a practical response to situations that mothers could not control, such as inadequate milk supply, illness, early infant separation, or work conditions. These results are consistent with the breastfeeding studies in Qatar and the Arab world, which persistently record a high breastfeeding intention, yet low exclusive and sustained breastfeeding rates compromised by biomedical, occupational, and social factors (53, 54). The introduction of formula, therefore, aligns with long-standing cultural norms, whereby breastfeeding is preferred. In cases where parents chose formula, their mentioned reasons were nutritional similarity to breast milk, digestive tolerance, infant acceptance, social observation, and personal investigations being auxiliary factors to the decision. On top of all these, relevant medical advice was essential, with HCPs reported as the main trustworthy source of information. Both the parent survey and the FG data showed that parents needed clinical approval, safety data, visible results in other children, and assurance of quality in manufacturing before they think of adopting HMO-enriched formula. The fear of industrial imitation of natural bioactive ingredients, the possibility of manufacturing mistakes, and the presence of unknown ingredients on the label, were indicative of probable precautionary disposition, in line with the findings of other studies in the Arab world especially when the product is aimed at a vulnerable population, such as infants (55, 56).

The perception score of HCPs and their previous encounters with HMOs and HMO-enriched formula were higher than those of parents. While most perception items were correctly recognized, there were gaps in answering areas related HMOs stability to stomach acidity and their availability in commercial infant formulas. The former gap may reflect an incomplete understanding of HMO bioavailability, and the latter may directly limit the potential of product-specific recommendations. Interestingly, even with moderate to high level of perceptions, fewer HCPs received formal training on HMOs, and almost half of them (47.5%) rated their own knowledge as fair. This knowledge limitation was supported during the FG discussions, when HCPs mentioned that they heard about HMOs incidentally rather than in a systematic manner. HMO-enriched formula perception was significantly related to recommending it to parents, whereas the objective perception score was not correlated with recommendation behavior. A parallel exploratory logistic regression model, therefore, may fit, with the recommendation of an HMO-enriched formula as the outcome and perception score and formal training on HMOs as possible associated factors.

However, the bivariate association between perception and recommendation should be interpreted alongside the small HCPs sample size, sparse training data, and under-specified model. The trend is consistent with the broader literature on HCPs recommendation behavior in infant nutrition. For instance, a study found that physicians’ recommendations on breastfeeding were more strongly correlated with their own exposure to breastfeeding information than with formal training (57), a finding replicated in subsequent studies on infant nutrition counseling (58). In a wider sense, a trend where familiarity, which is measured in terms of exposure and recognition, may be a more effective behavioral determinant than depth of understanding, has been reported in various clinical areas, where providers are more likely to counsel on what they know and feel comfortable about, rather than using their formal education (59). The current research contributes to this literature by introducing HMO-specific information and locating it in the Qatari setting.

The above quantitative patterns can be viewed in the context provided by the FG discussions. HCPs were concerned with infant formula caloric density, protein content, and iron and vitamin D levels, but not on prebiotic or oligosaccharide profiles. This possibly indicates the need to stress HMOs concepts in clinical nutrition education. HCPs recognized the theoretical feasibility of HMO supplementation and had conditional willingness to prescribe HMO-enriched formula, but cited the lack of confidence, knowledge, and explicit guidelines, and which could be possibly attainable through targeted educational interventions.

Integration of the quantitative and qualitative findings from this study revealed that perceptions of HMOs and HMO-enriched formula were limited among parents, while healthcare providers had greater familiarity but less formal training. Although both groups expressed willingness to use or recommend these products, the qualitative data clarified that such conditional acceptance depended on safety assurance, credible evidence, affordability, and trusted professional guidance. The integrated results, therefore, suggest that HCP-led education and counseling of parents may be essential to support informed decision-making and potential willingness to use HMO-enriched formula in Qatar.

Some implications for the current findings in practice and research can be conferred; yet, the findings should be interpreted within the “breastfeeding-first” framework described above. Human milk remains the gold standard for infant nutrition and the principal HMOs source, and no infant formulas should be attempted at its replacement. While some participants expressed willingness to consider or recommend HMO-enriched formulas, this does not imply equivalence with breastfeeding; rather, these responses highlight areas where additional education, support, and protection of breastfeeding may be relevant. The introduction of structured continuing education programs to HCPs involved in infant care, on HMOs, and HMO-enriched formula, and practical advice to parents, would be an added value to professional development. In addition, HMO-enriched formula may constitute a good addition to national infant feeding recommendations in Qatar,. Parent education on HMOs can be a key method for raising awareness, alongside social platforms that a large percentage of parents already access for infant nutritional information. Health authorities and insurance companies ought to consider ways in which the accessibility and affordability of HMO-enriched formula become available to families with indicated formula requirements. Moreover, future studies that provide formal psychometric validation of HMOs perception score are reasonable. While the current study was cross-sectional, more comprehensive and longitudinal research that monitors perception and willingness to use over time would allow causal pathways to be explored. Similarly, intervention studies on the effects of HCPs training programs or parent education campaigns on perceptions and willingness to use would be essential to obtain critical evidence regarding the effectiveness of the approaches suggested here. The cost-effectiveness, cultural adaptation of communication strategies, and the particular channels through which parents can get HMOs information are also intriguing for investigation, and may help to create e models that can possibly be used as a baseline for education expanded to other healthy biological compounds as well.

## 5 Strengths and limitations

This study has several strengths, including, first, its novelty in Qatar and the Arab world to address HMO-specific perceptions and willingness to use in both parents and HCPs concurrently; the latter represented different specialties mainly working in governmental hospitals, indicating a clinically diverse and relevant institution-based workforce. Second, the mixed-methods design entails a specific methodological opportunity to offer representativeness and breadth through surveys, followed by contextual depth, explanatory affluence, and opinions of participants vocalized through FGs, bringing together an information landscape which could not be sufficiently produced by either method alone. Third, the parent survey was conducted in both Arabic and English, making it linguistically accessible to the multinational parent population of Qatar and minimizing exclusion bias that can be a significant issue with health surveys in multicultural environments. Fourth, the sample of HCPs was multidisciplinary, including pediatricians, dietitians, nurses, breastfeeding consultants, specialists, and midwives, warranting a multidisciplinary view. Fifth, rigorous non-parametric statistical tests were used to improve the reliability of the inferential analysis, whereas qualitative data inductively provided depth and context to many of the survey findings.

The findings of this study, however, are to be interpreted in light of several shortcomings. To begin with, we acknowledge that the sample sizes obtained were smaller than the pre-study estimates of both populations, and, the FG participants were not many. Specifically, in the qualitative component, FG discussions continued until no new themes or codes emerged across successive sessions within each participant group, indicating that thematic saturation was reached within the sample obtained, yet with a smaller sample than initially planned. Although saturation was considered achieved, the small and geographically restricted qualitative sample may limit transferability of the findings to other settings or contexts. Also, the deficit was especially high for HCPs participating in the survey, with the ultimate sample about 44% lower than required. This limitation persisted despite repeated attempts to collect more responses and multiple reminders to engage both groups, and could be understandable especially for HCPs given their numerous commitments and high workload. As recruitment was done online and voluntarily, with substantial nonresponse and exclusions, the participants may be more engaged, educated, or interested than the target population, limiting the generalizability of the results. The limitation in statistical power makes the risk of not finding real associations more likely, especially in the HCP analyses.

Furthermore, although convenience sampling was used to facilitate recruitment and reduce logistic challenges in this exploratory mixed-methods investigation, it may have introduced selection bias and limited the representativeness of the study population. Because convenience sampling rather than probability-based sampling was employed, the study population may not fully represent all parents and HCPs in Qatar, including the latter who work in the private sector. Participants who voluntarily responded to the survey may differ systematically from non-participants with respect to awareness, health literacy, motivation, or interest in infant nutrition, potentially introducing volunteer and selection bias. Consequently, the findings should be interpreted cautiously and should not be generalized to the wider Qatari population without further confirmation through larger, more representative, and multi-center studies employing probability sampling techniques.

The low response rate among HCPs also creates nonresponse bias: respondents might not represent the overall population, which may overstate or understate the estimated levels of perceptions, or may underreport the actual level of professional training. As such, a more comprehensive study with improved sample size would contribute to more representative results and better understanding of the topic in the context of Qatar.

Second, and because no validated HMO perception instrument currently exists, the seven-item HMO perception index was specifically developed for this exploratory study based on the available literature and pilot testing. Although the items demonstrated face validity and covered important domains of HMO knowledge, the observed Cronbach’s α coefficients indicated low internal consistency, confirming that the index should not be interpreted as a psychometrically validated unidimensional scale. Rather, it should be viewed as an exploratory descriptive knowledge index. Consequently, all analyses involving this index should be interpreted cautiously and considered hypothesis-generating rather than confirmatory or inferential. Future studies should develop and formally validate comprehensive HMO perception instruments using established psychometric procedures, including exploratory and confirmatory factor analysis, test–retest reliability, construct validity, and criterion validity.

There is also a possibility that some of the items were prone to correct answers through guessing on a true/false basis, which can artificially inflate scores and restrict the accuracy of our assessment. Should the validity and reliability of the scale be formally tested in a future study, this would permit more accurate and comparable evaluation of perceptions of HMOs among the target population. Third, the quantitative component is cross-sectional, and causal inference is not possible. The reported relationships between perceptions and willingness to use are correlational by nature, and directional assertions cannot be supported without longitudinal or experimental evidence. It is also quite reasonable that a greater willingness to use HMO-enriched formula is a cause of greater information-seeking and perception, but not the other way round. Although exploratory logistic regression models were used to examine factors associated with willingness to use or recommend HMO-enriched formula, the cross-sectional design precludes causal inference. Consequently, the reported odds ratios should be interpreted as measures of association rather than evidence of causal or temporal relationships. Although exploratory subgroup analyses were undertaken where feasible, the modest sample size within some healthcare provider subgroups limited statistical power. Larger multicenter studies are needed to investigate potential differences across healthcare sectors and professional settings, including HCPs in the private sector in Qatar. Fourth, the use of self-reported data presents the risk of recall bias, social desirability bias and inconsistency of responses. Fifth, our instruments did not include explicit questions requiring participants to distinguish HMOs from probiotics, prebiotics, or general dietary supplements. As such, residual misunderstanding cannot be excluded, and the findings reflect participants’ responses to HMOs as provided in the study, rather than systematically verified knowledge of the true distinctions among these product categories. Sixth, FG member checking was not conducted, as participants were not available for follow-up; this is acknowledged as a limitation of the qualitative component. To mitigate this, credibility was rather supported through verbatim participant quotations, systematic and team-reviewed coding, and triangulation with the quantitative findings, as described in the Methods.

## 6 Conclusion

Overall, the current study presents an initial description of perceptions of HMOs and HMO-enriched formula among parents and healthcare providers in Qatar. The findings indicate variable awareness, limited formal training among healthcare providers, and cautious attitudes toward use, highlighting important informational gaps. Generally, low proportion of parents had heard about HMO and HMO-enriched formula, alongside a moderate perception score and cautious willingness to use; relatively greater HCPs heard about HMOs, but with lower formal training and variable recommendation patterns. The combination of quantitative and qualitative results reveals an explanatory background: perception may be associated with greater engagement, while trust and practical barriers may shape how HMO-enriched formula is considered and used. Because the HMO perception index has not yet undergone formal psychometric validation, all findings involving this exploratory index should be interpreted cautiously and regarded as hypothesis-generating pending confirmation using validated measurement instruments. While the study identified some patterns of awareness, perceptions, and information needs among parents and HCPs in Qatar, it does not directly demonstrate that improved awareness will directly increase adoption of HMO-enriched formula or improve infant health outcomes. Future multicenter studies employing probability-based sampling strategies and larger representative samples are needed to validate these findings and improve their generalizability to the wider population in Qatar and similar settings.

## Data Availability

The datasets presented in this article are not readily available because of privacy of the participants. Reasonable requests to access the datasets that do not breach participant privacy and comply with national and institutional guidelines should be directed to dhammoude@qu.edu.qa and ahmed.malki@qu.edu.qa.
