# Supplementary material for: Exploration of parents’ and healthcare providers’ perceptions on human milk oligosaccharides (HMOs) and willingness to use HMO-enriched formula: a baseline mixed-methods study from the State of Qatar
Source: Front Nutr. 2026 Sep 23;13:1909697. doi: 10.3389/fnut.2026.1909697 (PMC13642783; doi:10.3389/fnut.2026.1909697)
Supplement: Supplementary file 1 [file Table_1.DOCX]

Supplementary Material

# 1 Supplementary Table 1

Qatari vs. Non-Qatari parents survey outcome comparisons

| **Variable** | **Category** | **Qatari** | **Non-Qatari** | **Test** | **Statistic** | **P-value** | **FDR-adjusted p** | **Effect size** | **Effect size type** |
| --- | --- | --- | --- | --- | --- | --- | --- | --- | --- |
| **Breastfeeding experience** | No | 12 (8.7%) | 5 (9.8%) | Fisher's exact | - | 0.780 | 0.813 | 0.017 | Cramér's V |
|  | Yes | 126 (91.3%) | 46 (90.2%) |  |  |  |  |  |  |
| **Current feeding pattern** | Exclusive breastfeeding | 35 (25.4%) | 18 (35.3%) | Chi-square | 3.395 | 0.183 | 0.540 | 0.134 | Cramér's V |
|  | Exclusive formula feeding | 41 (29.7%) | 9 (17.6%) |  |  |  |  |  |  |
|  | Partial breastfeeding with formula | 62 (44.9%) | 24 (47.1%) |  |  |  |  |  |  |
| **Preferred information source about HMOs** | Formula packaging | 5 (3.6%) | 3 (5.9%) | Fisher's exact (Freeman-Halton) | - | 0.398 | 0.540 | 0.163 | Cramér's V |
|  | Friends and family | 5 (3.6%) | 3 (5.9%) |  |  |  |  |  |  |
|  | Healthcare provider | 49 (35.5%) | 20 (39.2%) |  |  |  |  |  |  |
|  | Other/ No preferences | 3 (2.2%) | 0 (0.0%) |  |  |  |  |  |  |
|  | Scientific literature | 34 (24.6%) | 16 (31.4%) |  |  |  |  |  |  |
|  | Social media/internet | 42 (30.4%) | 9 (17.6%) |  |  |  |  |  |  |
| **Interest in knowing more about HMOs** | No | 15 (10.9%) | 2 (3.9%) | Fisher's exact | - | 0.164 | 0.540 | 0.108 | Cramér's V |
|  | Yes | 123 (89.1%) | 49 (96.1%) |  |  |  |  |  |  |
| **Should HMO-enriched formula exist/be used in Qatar** | Strongly agree/ Agree | 84 (60.9%) | 37 (72.5%) | Fisher's exact (Freeman-Halton) | - | 0.288 | 0.540 | 0.114 | Cramér's V |
|  | Neutral | 49 (35.5%) | 12 (23.5%) |  |  |  |  |  |  |
|  | Disagree/ strongly disagree | 5 (3.6%) | 2 (3.9%) |  |  |  |  |  |  |
| **Would recommend to a mother who cannot breastfeed exclusively** | No | 10 (7.2%) | 3 (5.9%) | Fisher's exact (Freeman-Halton) | - | 0.813 | 0.813 | 0.047 | Cramér's V |
|  | Not sure | 70 (50.7%) | 24 (47.1%) |  |  |  |  |  |  |
|  | Yes | 58 (42.0%) | 24 (47.1%) |  |  |  |  |  |  |
| **HMO-enriched formula recommended by HCP in Qatar** | No | 38 (27.5%) | 8 (15.7%) | Chi-square | 10.745 | 0.005 | 0.060 | 0.238 | Cramér's V |
|  | Not sure | 90 (65.2%) | 31 (60.8%) |  |  |  |  |  |  |
|  | Yes | 10 (7.2%) | 12 (23.5%) |  |  |  |  |  |  |
| **Preferred formula to purchase** | HMO -enriched formula | 47 (34.1%) | 21 (41.2%) | Chi-square | 1.998 | 0.368 | 0.540 | 0.103 | Cramér's V |
|  | No specific preferences | 70 (50.7%) | 20 (39.2%) |  |  |  |  |  |  |
|  | Regular formula | 21 (15.2%) | 10 (19.6%) |  |  |  |  |  |  |
| **Effect of HMO addition on formula choice** | I am not sure | 68 (49.3%) | 20 (39.2%) | Chi-square | 1.756 | 0.416 | 0.540 | 0.096 | Cramér's V |
|  | No, it will not affect my choice | 26 (18.8%) | 10 (19.6%) |  |  |  |  |  |  |
|  | Yes, I will be more likely to choose it | 44 (31.9%) | 21 (41.2%) |  |  |  |  |  |  |

# 2 Supplementary Table 2

# Integration of qualitative and quantitative findings (presented as a Joint Display Table, as referenced in the Data Integration section of the Results)

| **Quantitative Phase** | | | | **Qualitative Phase** | | |
| --- | --- | --- | --- | --- | --- | --- |
| **Parents’ and healthcare providers’ perceptions on HMOs and willingness to use HMO-enriched formula** | | | | | | |
| **Domain** | **Summary statistic** | **Item** | **Quantitative finding** | **Theme** | **Sub-theme** | **Quote** |
| **Aligning results** | | | | | | |
| **Parent breastfeeding experience** | 91.0% yes | Parents breastfed previously | Most parents reported previous breastfeeding experience. | Theme 1. Infant feeding experiences and perceived challenges | Sub-theme 1.1: The primacy of breastfeeding and barriers to sustaining it | *"The challenge is how to keep the milk flowing and ensure it meets the baby’s needs." (Speaker 1)* |
| **Parent current feeding pattern** | 45.5% partial breastfeeding with formula | Parent current nutrition | Partial breastfeeding with formula was the most common current feeding pattern, followed by exclusive breastfeeding and exclusive formula feeding. | Theme 1. Infant feeding experiences and perceived challenges | Sub-theme 1.2: Circumstances leading to formula introduction | *"Formula is only coverage during work hours, not a healthy alternative." (Speaker 1)* |
| **Parent formula selection priorities** | 36.0% preferred HMO-enriched formula; 47.6% no specific preference | Preferred formula if needed | Many parents had no specific formula preference, while over one-third preferred HMO-enriched formula if needed. | Theme 2. Factors shaping infant formula selection | Sub-theme 2.1: Nutritional proximity to breast milk and digestive tolerance | *"The most important thing I look for is something as close as possible to breast milk; I know nothing can match it, but that is what I am looking for." (Speaker 2)* |
| **Parent information source** | 36.5% healthcare provider | Preferred information source | Healthcare providers were the most preferred source of information among parents. | Theme 2. Factors shaping infant formula selection | Sub-theme 2.2: Role of medical guidance, social influence, and personal experience | *"I relied on the experiences of those around me and some reading." (Speaker 8)* |
| **Parent having heard about HMOs** | 19.0% yes; 64.0% no | Heard about HMOs | Only 19.0% of parents had heard about HMOs, while 64.0% had not heard and 16.9% were unsure. | Theme 3. Limited perception but openness to learning about HMOs | Sub-theme 3.1: Limited prior awareness of HMOs | *"I have no idea- this is the first time I have heard about it." (Speaker 1)* |
| **Parent interest in learning** | 91.0% yes | Interested in knowing more about HMOs | Most parents expressed interest in learning more about HMOs. | Theme 3. Limited perception but openness to learning about HMOs | Sub-theme 3.2: Engagement with HMOs information during discussion | *"A substance with this importance, we must be educated about it, because we are hearing about it for the first time." (Speaker 1)* |
| **Parent willingness to use HMO-enriched formula** | 18.5% yes; 62.4% possibly | Will use HMO-enriched formula | Most parents selected “possibly,” indicating cautious rather than definite willingness. | Theme 4. Perceptions of and willingness to use HMO-enriched formula | Sub-theme 4.2: Conditions and perceived benefits that may facilitate use | *"I would use it only if recommended by a trusted doctor... someone who has seen the evidence and is confident enough to tell me it is safe for my child." (Speaker 7)* |
| **HCP having heard about HMOs** | 75.4% yes | Heard about HMOs | Most HCPs had heard about HMOs. | Theme 2. Perceptions of HMOs | Sub-theme 2.1: Perceptions gaps levels | *"We, truly speaking, don’t know much about it... human milk has high HMOs... It’s important for immunity." (Speaker 10, Pediatric Consultant)* |
| **HCP functional perception of HMOs** | 91.8–95.1% for key benefit items | HMO benefit perception items | Correct responses were high for major HMO benefits, including natural presence in human milk, intestinal flora, immunity, and allergy-related benefit. | Theme 2. Perceptions of HMOs | Sub-theme 2.2: Functional understanding of HMOs role in gut health | *"Oligosaccharides support the baby’s immune system and prevent infections." (Speaker 5, Nurse)* |
| **HCP willingness to recommend** | 82.0% yes | Recommend HMO-enriched formula to mothers who cannot breastfeed exclusively | Most HCPs indicated willingness to recommend HMO-enriched formula in this context. | Theme 5. Willingness to recommend HMO-enriched formula | Sub-theme 5.2: Perceived benefits of HMO-enriched formula | *"If synthesized HMOs are added to baby formula, it will definitely benefit... But we need to know more... before we can recommend it to parents and families." (Speaker 10, Pediatric Consultant)* |
| **Expanding results** | | | | | | |
| **Parent clinical communication gap** | 64.0% unsure | Are HMO-enriched formulas recommended by HCPs in Qatar? | Most parents were unsure whether HCPs recommend HMO-enriched formula; 24.3% reported that it was not recommended. | Theme 2. Factors shaping infant formula selection | Sub-theme 2.2: Role of medical guidance, social influence, and personal experience | *Mothers relied on healthcare advice, family and peer experiences, social observation, and their own reading when choosing formula.* |
| **HCP formal training** | 13.1% yes | Formal training on HMOs | Only 13.1% of HCPs reported receiving formal training on HMOs despite general awareness. | Theme 2. Perceptions of HMOs | Sub-theme 2.3: Limited perceptions of HMOs in Qatar’s community in HCPs’ opinion | *"Even as we are working in this facility, we know we are still not confident about it. We don’t have enough information about the HMOs." (Speaker 12, Registered Midwife)* |
| **HCP advising behavior** | 41.0% yes | Ever advised HMO-enriched formula | Less than half of HCPs had advised HMO-enriched formula. | Theme 3. Use of HMO-enriched infant formula | Sub-theme 3.1: HMO-enriched formula recommendation | *"Truly speaking, we don’t know much. We don’t know enough to recommend." (Speaker 10, Pediatric Consultant)* |
| **HCP focus during formula selection** | Clinical-practice pattern | Formula counselling priorities | HCPs reported that clinical counselling tends to focus on the overall formula profile rather than HMO content specifically. | Theme 3. Use of HMO-enriched infant formula | Sub-theme 3.2: Prioritizing other components in infant formula | *"I will look at the protein itself, whether it is hydrolyzed or not." (Speaker 1, Breastfeeding Specialist)* |
| **HCP having heard about HMOs and advising** | p < 0.001; V = 0.511 | Heard about HMO-enriched formula vs advised formula | HCP awareness of HMO-enriched formula was strongly associated with having advised it to parents. | Theme 3. Use of HMO-enriched infant formula | Sub-theme 3.1: HMO-enriched formula recommendation | *"In general, we never focus on oligosaccharides. We never do." (Speaker 6, Neonate Dietitian)* |
| **HCP cost barrier** | Multiple barriers | Barriers to parental purchase | Cost was identified as one of the practical barriers that may limit uptake. | Theme 4. Challenges in recommending HMO-enriched infant formula | Sub-theme 4.1: Cost and affordability | *"If it is more costly, parents will not be able to afford it." (Speaker 9, NICU Dietitian)* |
| **HCP having heard about HMOs and availability barrier** | Multiple barriers | Barriers to parental purchase | Limited professional awareness and market availability were identified as barriers to recommendation and uptake. | Theme 4. Challenges in recommending HMO-enriched infant formula | Sub-theme 4.2: Lack of professional perceptions and market availability | *"Even healthcare providers are not aware about that... awareness and availability are important." (Speaker 3, Breastfeeding Specialist)* |
| **HCP safety and tolerance barrier** | Multiple barriers | Barriers to parental purchase | Safety, allergy risk, taste, texture, and infant tolerance were raised as possible concerns. | Theme 4. Challenges in recommending HMO-enriched infant formula | Sub-theme 4.3: Safety concerns and tolerance | *"Allergy is one of the things we should look for. Also, tolerance... how these complexes might change the taste and texture of the formula." (Speaker 11, Pediatric Resident)* |
| **Contrasting results** | | | | | | |
| **Parent support vs uncertainty** | 64.1% support; 32.3% neutral | Availability in Qatar vs uncertainty | Although most parents supported availability, nearly one-third remained neutral. | Theme 4. Perceptions of and willingness to use HMO-enriched formula | Sub-theme 4.1: Safety concerns and barriers to acceptance | *"It is an imitated substance... I do not know what guarantees me that they have put the correct formulation." (Speaker 1)* |
| **Parent willingness vs perception score** | p = 0.295 | Perception score across willingness categories | Perception score alone was not significantly associated with willingness to use HMO-enriched formula. | Theme 4. Perceptions of and willingness to use HMO-enriched formula | Sub-theme 4.2: Conditions and perceived benefits that may facilitate use | *"Reliable studies and trials, not just marketing." (Speaker 8)* |
| **HCP having heard about HMOs vs formal preparation** | 75.4% heard; 13.1% trained | Awareness and formal training | Most HCPs had heard about HMOs, but few had received formal training. | Theme 2. Perceptions of HMOs | Sub-theme 2.1: Perceptions gaps levels | *"Most of the questions of the survey about HMOs I answered ‘I don’t know’... I don’t have that level of knowledge." (Speaker 2, Breastfeeding Specialist)* |
| **HCP willingness vs practice** | 82.0% willing; 41.0% advised | Willingness to recommend vs previous advising behavior | Most HCPs were willing to recommend HMO-enriched formula, but fewer had advised it in practice. | Theme 5. Willingness to recommend HMO-enriched formula | Sub-theme 5.1: Irreplaceability of breastfeeding | *"Mother’s milk is designed according to the baby’s requirements and cannot be substituted." (Speaker 3, Breastfeeding Consultant)* |
